# Effects of Choice Set Sizes and Moderations of Anxiety and State Emotions on Mental Health Self-Care Uptake, Engagement, and User Experience: Experimental Study

**DOI:** 10.2196/71165

**Published:** 2025-12-15

**Authors:** Siu Kit Yeung, Florence H T Leung, Jason C M Lee, Gabriel Man Hin Cheung, Ching Wan Li, Winnie W S Mak

**Affiliations:** 1 Department of Psychology, The Chinese University of Hong Kong, Shatin, N.T., Hong Kong SAR, China (Hong Kong), 852 3943 6577

**Keywords:** digital mental health, decision making, choice set size, individual moderators, anxiety, state emotions

## Abstract

**Background:**

Digital mental health platforms often consist of many different forms of self-care exercises. To our knowledge, whether the number of choices presented to the users affects their uptake and experiences and poses negative consequences (ie, not choosing any exercises, choice dissatisfaction) for users, especially those experiencing anxiety and depressive symptoms or unpleasant state emotions, has not been empirically investigated.

**Objective:**

This study investigated the impact of choice set size on practice decisions, completion, satisfaction, and subjective experiences, as well as potential moderators including depression and anxiety symptoms, state emotions, and motivational and decisional attributes on these choice outcomes.

**Methods:**

Participants were recruited through university mass email and social media, and 652 participants were included in our analyses. Participants completed questions regarding anxiety and depressive symptoms, state emotions, and other psychological attributes. Then, they were randomly assigned to 1-choice, 4-choice, and 16-choice conditions, in which they may choose a self-care activity to practice or decide not to practice. Finally, they completed questions regarding completion, satisfaction, engagement, attitude, and perceived improvement in psychological state.

**Results:**

Presenting multiple choices resulted in a higher likelihood of practice (odds ratio 3.12, 95% CI 2.08 to 4.67 and 3.83, 95% CI 2.55 to 5.76; *P*<.001) and better decision satisfaction (16-choice vs 1-choice: *d*=0.36, 95% CI 0.17 to 0.56, *P*<.001; 4-choice vs 1-choice: *d*=0.24, 95% CI 0.05 to 0.43, *P*=.03) compared with presenting with a single choice. Tentative evidence indicates anxiety symptoms and state emotions were meaningful moderators. Specifically, for individuals with more anxiety symptoms and intense negative emotions, presenting a larger choice set (16 choices) resulted in more positive chosen exercise satisfaction, better attitudes toward chosen activity, and higher perceived improvement in mental health state after the activity, when compared with presenting with smaller choice sets (anxiety: *β*=−0.38, 95% CI −0.69 to −0.06 to −0.51, 95% CI −0.84 to −0.18; state emotions: *β*=−0.31, 95% CI −0.66 to 0.03 to −0.60, 95% CI −0.92 to −0.28). No evidence was found for the moderating effect of motivational and decisional attributes.

**Conclusions:**

The moderation results were contradictory to prior research and our expectation that a larger choice set may result in worse outcomes than a smaller choice set for people who were experiencing higher levels of psychological distress. We speculated that a possible reason for these findings may be that people with more anxiety symptoms and unpleasant emotions may have a stronger need to reduce these uncomfortable symptoms and emotions, and when presented with more choices on self-care activities, there may be a higher possibility that these self-care activities can address their distress.

## Introduction

### Background

To maintain and enhance positive mental health, practicing mental health self-care regularly is essential in our daily lives. With the proliferation of digital mental health apps and platforms, numerous exercises derived from approaches such as cognitive-behavioral therapy and mindfulness-based interventions [1,2] become easily accessible. These activities can be guided through audio or visual instructions or completed using worksheets. While having more exercises available may cater to different needs of service users, presenting too many choices on the platform may negatively influence the likelihood of practice, engagement, and satisfaction, especially for people experiencing anxiety and depressive symptoms or unpleasant emotions [1]. Are users more motivated to do mental health self-care exercises, and are their experiences more satisfactory and engaging if they are given fewer or a larger number of choices? Moreover, are these choice decisions and the related completion of practices, engagement, satisfaction, and experiences moderated by users’ present psychological distress, emotional states, and individual differences in motivation and decision-making?

While numerous studies have explored the effects of different choice quantities in economic and consumer decision-making contexts [3-7] and some digital health studies have investigated the efficacy of choice provision versus the absence of choice or tunneling [8-10], research comparing a higher number of choices over a low number of choices in health-related settings is scarce. Only a handful of studies have addressed this comparison in health-medical contexts [7,11,12], and to our knowledge, there is no such study on digital mental health self-care. Making choices for mental health self-care is different from many other consumer behaviors. In previous research, consumer behaviors often involve commercial transactions over the buying of daily goods (ie, pens, jam), with multiple dozens of choices in the large choice set [4]. Often, when research involves making health-oriented decisions, the number of choices is generally lower (under 20 choices) compared with other consumer behavior domains [11,12]. Unlike other consumer behaviors, these choices may have more bearing on well-being and health and may be considered as more consequential than daily goods choices for most people. With the increasing popularity of digital mental health, research on choices of mental health self-care may have informative implications for personalization of self-care offerings and understanding of the public’s choices over mental health-related self-care activities.

### More Choices-Are-Better Perspective

Multiple theories in psychology and economics suggest that individuals tend to experience greater satisfaction when presented with multiple choices compared with having only one option available or a limited number of choices. The availability of multiple choices is believed to support feelings of autonomy, a fundamental human need that boosts motivation based on Self-Determination Theory [5,13,14]. Relatedly, having multiple choices can enhance perceptions of freedom in decision-making and elevate choice satisfaction [3,4,15]. Such beneficial effects have been observed in some health-related contexts such as illness screening and physical exercise [16]. Classical economic models also suggest that a higher number of choices increases the likelihood of individuals identifying and selecting options that match their personal preferences and goals [3,4,17,18].

However, with Chinese cultures emphasizing a culture of deference and compliance to authority [19,20] while Chinese people generally have lower mental health literacy [21,22], but higher levels of mental illness stigma [21,23], they may prefer being told which choice to opt for or may benefit more from a smaller choice set in terms of mental health self-care (see studies by Chernev et al [4], Misuraca et al [5], and Mogilner et al [24] regarding the association between lower expertise/familiarity and better outcomes with a smaller choice set). Thus, understanding how Chinese people respond to different choice sets in a digital mental health platform for self-care under a mix of this theoretical and cultural backdrop has important implications for how researchers and mental health practitioners could promote public mental health in Chinese societies.

### Overchoice Effect or Too-Many-Choices Perspective

Contrary to traditional economic beliefs that more choices lead to better outcomes, some studies over the past 2 decades have indicated that an abundance of choices might diminish the likelihood of purchase and dampen choice satisfaction under certain circumstances [4,25-27]. Some individuals may find themselves overwhelmed by an excess of options, leading to decision-making challenges and dissatisfaction due to limitations or exhaustion in cognitive resources and capabilities [5,26,28]. Being presented with many choices, individuals may experience an increase in cognitive costs in processing more information, making judgments and decisions [4]. This phenomenon of negative repercussions from an excessive array of choices is often referred to as “choice overload” or “overchoice” [7,29,30]. However, while numerous studies, meta-analyses, and reviews have investigated this topic, the findings remain mixed, sometimes inconclusive, and controversial [4,30]. The overchoice effect may manifest under specific conditions, such as increased decision complexity and choice set intricacy [4], and may affect individuals with certain psychological attributes, such as prevention regulatory focus [31] or in emotional states such as anxiety [5,32]. In the mental health realm, for individuals with anxiety or depressive symptoms and state unpleasant emotions, it is worthwhile to examine how choice autonomy, which aligns with personal recovery and self-determination, and cognitive overload, which may be particularly relevant for people experiencing psychological distress, may affect decisions, engagement, and experiences with different choice set sizes.

### Anxiety, Depression, and State Emotions as Potential Moderators

One of the individual difference moderators that may be relevant is the experience of anxiety. Studies by Hu et al [32] found that individuals experiencing high levels of anxiety were more prone to facing decision difficulties and delaying choices when confronted with a larger array of options. Hu et al [32] posited that anxiety demands more cognitive resources, thereby reducing the capacity for processing information and rendering judgments based on more information within larger choice sets, so a lower number of choices may be preferred by people experiencing high levels of anxiety. This heightened challenge when encountering a larger choice set in decision-making might lead individuals to postpone choices or not choose any option and perhaps experience lower satisfaction [32-34].

Related to but distinct from anxiety, we are not aware of any study on the potential moderating effect of depression in choice set size effects. Existing research indicates that many individuals experiencing depression often contend with fatigue [35,36] and may experience amotivation [36]. Similar to cognitive resource limitations among individuals with anxiety [32], people with depression may find it challenging to process information involving more choices due to diminished energy and motivation levels. Individuals with depression may avoid activities that demand extensive cognitive resources [37-39] or encounter difficulties and unpleasant experiences when engaging in complex tasks or decisions that require heightened cognitive engagement [38,40,41]. Thus, when the number of choices is high, one may anticipate that individuals exhibiting more depressive symptoms may be more likely to experience the overchoice effect.

Although the above-discussed studies about anxiety and depression were conducted outside the digital mental health context, concerns have been raised by digital mental health researchers regarding the potential negative impact of offering users a wide array of choices, suggesting the possibility of triggering the overchoice effect [1,42,43]. Notably, Valentine et al [1] contended that individuals with depression and anxiety disorders may encounter cognitive challenges, such as difficulties in concentration, rendering them more susceptible to experiencing the adverse effects of overchoice and disengagement when confronted with excessive information and numerous options. It is imperative to investigate whether limiting the number of self-care choices presented, particularly for individuals with heightened levels of anxiety and depression, would lead to better outcomes than presenting an extensive number of choices.

In addition to anxiety and depressive symptoms, individuals’ current emotional states may affect their choice preferences. Drawing from the rationale applied to anxiety and depressive symptoms, it is hypothesized that individuals reporting higher levels of anxiety or depressive symptoms, or stronger unpleasant emotions, would benefit more from a restricted number of choices (1 choice or 4 choices) compared with a larger choice set (16 choices), including greater uptake, engagement, and completion of the chosen activity as well as satisfaction with the decision.

### Tailoring the Number of Choices Based on Individual Differences in Regulatory Focus and Choice Preferences

In addition to testing the potential interactions between the number of choices and mental health conditions and emotional states, it may be valuable to investigate the moderating effects of dispositional individual characteristics. One pertinent characteristic worth considering is regulatory focus, as proposed by Higgins [44], which pertains to the motivational orientations in goal pursuits and decision-making processes. Regulatory focus can be categorized into prevention focus that emphasizes safety and the prevention of losses or errors, and promotion focus that emphasizes seeking advancements, gains, and growth opportunities.

Research by Tuan Pham and Chang [45] found that individuals with a predominant promotion focus tend to view options selected from a larger choice set more favorably than those from a smaller set. This inclination was attributed to the proactive nature of promotion-oriented individuals, who are eager to capitalize on opportunities that may be more abundant in wider choice sets [45]. Furthermore, promotion-focused individuals often aim to maximize gains and pursue idealistic goals, making them more likely to find an option that aligns with their preferences in a larger selection. Another related explanation is that promotion-focused individuals tend to strive for maximizing gains and are more idealistic [46,47], and a higher number of choices is more likely to consist of an ideal option that matches well with the preference of the decision maker [3,4,17]. In contrast, Polman [31] found that prevention-focused individuals are more affected by choice overload. They worry about making mistakes and are vigilant about “bad options.” With more choices, they perceive a higher risk of encountering these “bad options.” Therefore, presenting fewer choices may be better for prevention-focused individuals. Given the above findings, it is hypothesized that for predominantly promotion-focused individuals, presenting a larger choice set will be more beneficial than presenting a smaller choice set, whereas for predominantly prevention-focused individuals, presenting a smaller choice set will be better than presenting a larger choice set.

Apart from regulatory focus, a relatively simple potential method of choice-set-individual-characteristics tailoring was tested by directly asking participants their preferences and perceived difficulty in making decisions when given many choices. For people who prefer many choices and perceive making decisions as less difficult [48], we expect that a higher number of choices (19) will be more beneficial in enhancing their practice likelihood, completion rates, decision satisfaction, choice satisfaction, and engagement compared with a lower number of choices (1 choice or 4 choices). In contrast, for people who do not prefer many choices and people who perceive making such decisions as difficult, a lower number of choices (1 choice or 4 choices) may be better than 16 choices. Broadly speaking, accommodating or matching individual preference is generally associated with engagement, choice satisfaction, and completion rates in mental health contexts [49,50]. This method of tailoring may seem intuitive, simple, and feasible to implement if shown to be effective.

### Summary of Hypotheses

To summarize, we investigated both symptoms of psychological distress and state emotions, as well as psychological attributes as moderators. We hypothesized the following for practice likelihood, completion of exercise, satisfaction, and engagement:

- For people experiencing lower levels of anxiety, a higher number of choices (16) will result in better outcomes than lower numbers of choices (1 or 4). For people experiencing higher levels of anxiety, a lower number of choices (1 or 4) is expected to result in better outcomes than a higher number of choices (16).
- For people with fewer or no depressive symptoms, a higher number of choices (16) will lead to better outcomes than lower numbers of choices (1 or 4). For people experiencing more depressive symptoms, a lower number of choices (1 or 4) will result in better outcomes than a higher number of choices (16).
- For people with more intense pleasant emotions, a larger choice set (16) will result in better outcomes than a smaller choice set (1 or 4). For people experiencing more intense unpleasant emotions, a smaller choice set (1 or 4) will lead to better outcomes than a larger choice set.
- For people who are predominantly promotion-focused, a larger choice set (16) is more likely to result in better outcomes than a smaller choice set (1 or 4). For people who are more prevention-focused, a smaller choice set (1 or 4) will lead to better outcomes than a larger choice set (16).
- For people who prefer many choices and perceive making decisions as less challenging, a wider choice set (16) will lead to better outcomes than a smaller choice set (1 or 4). For participants who do not prefer many choices and perceive making decisions as more difficult, a smaller choice set (1 or 4) is more likely to result in better outcomes than a larger choice set (16).

## Methods

### Open Science Disclosures

We preregistered our 3-condition experiment in the Open Science Framework (OSF), with the template by Van’t Veer and Giner-Sorolla [51]. The preregistration document, data files, code, outputs, and materials of our study are available in OSF (Table S2 in Multimedia Appendix 1 [31,52-67] contains links).

### Sample Size Determination, Recruitment, and Participants

We are not aware of any empirical study regarding choice set size in digital mental health exercises or investigating individual difference moderators on the impact of choice set size in mental health decision-making and experiences. Thus, our power analysis was not based on effect sizes of prior studies. We determined the minimum sample size through power analysis based on an effect size of *f*=0.18 (a small to medium effect based on Cohen [68]) that we consider as practically meaningful to detect while considering resource constraints, monetary and time costs, and number of potential participants [69]. We conducted the power analysis with G*Power (version 3.1; Heinrich-Heine-Universität Düsseldorf) [70]. With an expected alpha of 0.00167 (0.05/30, since we expected to adjust alpha with Holm correction to account for the number of hypothesis testing), 3 conditions, and a power of 80%, a minimum sample size of 575 would be required. Accounting for potential attrition and exclusion of participants not meeting validity testing, our target sample size was set as 675.

We recruited participants through the university subject pool and the social media platforms (Facebook and Instagram). We stated in the advertisement and the introductory page of the study that participants should understand Chinese and should be 18 years or older. The number of participants who were randomized to one of the conditions (1-choice, 4-choice, or 16-choice conditions) was 675.

The number of participants who completed at least one dependent variable measure and passed at least one validity check question (thereby included in our analyzed sample) was 652 (1-choice: 216 participants, 4-choice: 220 participants, 16-choice: 216 participants). A total of 23 participants were excluded due to not completing the experiment or not passing at least one validity check. The mean age of the analyzed sample was 21.77, with a SD of 12.41. A total of 40.03% of participants were women, 57.66% were men, and 2.31% of participants did not disclose gender.

### Procedures and Measures

The randomized 3-condition experiment was conducted through Qualtrics. To measure pre-existing characteristics or states, participants first completed scales on anxiety and depressive symptoms, emotional states, and psychological attributes (regulatory focus, preferences, and difficulties in making choices). In mental health self-care, it is reasonable to first gauge participants’ distress experience (as measured by anxiety and depressive symptoms) and their present emotional state at the time of engaging in a mental health platform, as this may affect their decision-making and we may adjust based on these potential moderators. This also simulates digital mental health contexts in which pre-existing states or psychological attributes are measured for personalization purposes. The effects of different choice set sizes on emotional states, anxiety, or depression were not tested, so such measures were presented before choice presentations only. Participants were randomized to either 1-choice, 4-choice, or 16-choice conditions, in which they chose to practice with one of the mental health exercises or chose not to practice. After that, participants responded to outcome variables including satisfaction, engagement, and subjective experiences. The entire experiment takes around 20 to 25 minutes. Details about the scales and measures are reported in the following.

### Choice Set Sizes

#### Rationale

In the context of mental health self-care, we anticipated a possibility that for a proportion of participants experiencing moderate to severe levels of depression, they may be amotivated to make choices on their own [36]. Moreover, in the Chinese culture, many people were socialized since very young to be deferent to authorities and exercise conformity or compliance [19,20]; thus, some Chinese people may find making choices more challenging or prefer simply to be told what to do. As such, we included a single-choice option. We are aware that the choice set size literature generally focuses on comparing various levels of choice sets with a smaller number of multiple choices to a very large number of choices (meta-analysis by [4]); however, we are unaware of any study that focused on choice set sizes for mental health-related self-care exercises. In light of the clinical and cultural considerations, we decided to include a single-choice condition to investigate whether providing a single choice may be better for a subgroup of participants. Participants can still opt out of not practicing self-care, even with this single-choice option.

As to our decision of including 4-choice and 16-choice as the other 2 experimental conditions for investigation, we acknowledged that these choice set sizes contain fewer options than some consumer behavior studies that involve products such as pens and DVDs [4] and the decision does not have much implication for their health. However, our choice set sizes were similar to studies that involve health decision making and a study that found support for the overchoice effect among people with higher levels of anxiety (eg, [12,32,71], also see Chernev [4] meta-analysis regarding the number of choices in different studies). Health-related decisions generally involve less familiar and more complex information compared with consumer decisions like buying food and other daily life products. A 4-choice condition is considered a relatively manageable size, similar to other studies using smaller choice sets [12,32]. As to a larger choice set, presenting participants with 30 or more choices on mental health self-care appears unrealistic and may not even be appropriate in light of the generally low levels of mental health literacy and unfamiliarity of the public with mental health self-care among Chinese [21,22]. Moreover, considering the user interface of presenting various choices on mobile apps, the maximum number of presenting a 16-option choice set is deemed more suitable in real-world implementation. Although the exact number of choices is not definite, we deemed comparing a single, manageable few, and relatively large choice sets in digital mental health contexts to be realistic and meaningful, as any of these choice set sizes has potential implementation implications on a mental health mobile app. Due to research constraints (eg, number of participants to be recruited, funding), we could not include more than 3 conditions with a greater number of choices.

#### Anxiety Symptoms

Participants completed the Generalized Anxiety Disorder-7 (GAD-7) [72], which measures anxiety symptoms over the past 2 weeks and consists of 7 items with 0 to 3 Likert scales (0=Not at all, 1=Several days, 2=More than half the days, 3=Nearly every day). Sum scores were used, with higher total scores indicating more severe levels of anxiety symptoms. GAD-7 has been validated in both general and clinical populations and has shown adequate internal consistency, construct validity, and convergent validity [72-74]. With our sample, GAD-7 has an internal consistency of *α*=.922.

#### Depressive Symptoms

Depressive symptoms over the past 2 weeks were measured using the Patient Health Questionnaire (PHQ-9) [75], with 9 items and 0 to 3 Likert scales (0=Not at all, 1=Several days, 2=More than half the days, 3=Nearly every day). The score is the sum of all items, and higher scores indicate more severe levels of depressive symptoms. PHQ-9 has been validated in both clinical populations and general populations and has demonstrated good reliability, construct validity, and convergent validity [76,77]. In our sample, PHQ-9’s internal consistency was *α*=.876.

#### State Emotions

Apart from measuring anxiety and depressive symptoms, state emotions were measured by using 8 emotions with 2 emotions from each quadrant of the Circumplex Model [78]. The emotions measured include tired, sad (unpleasant and lower arousal), tense, distressed (unpleasant and higher arousal), happy, excited (pleasant and higher arousal), relaxed, and calm (pleasant and lower arousal). 5-point scales adapted based on The Positive and Negative Affect Schedule [79] were adopted (1=very slightly or not at all, 2=a little, 3=moderately, 4=quite intense, 5=very intense, slight changes in wording for 4th and 5th point). The internal consistency of the pleasant emotions subscale was *α*=.708, and the unpleasant emotions subscale was *α*=.834 in our study. For the analyses, we averaged the pleasant emotions and the unpleasant emotions to calculate composite scores for pleasant emotions and unpleasant emotions. We then calculated the difference between pleasant emotions and unpleasant emotions, in which a positive score implies more intense pleasant emotions (compared with unpleasant emotions), whereas a negative score implies more intense unpleasant emotions (compared with pleasant emotions).

#### Regulatory Focus

Promotion and prevention orientations in regulatory focus were measured using Gomez et al [80] Health Regulatory Focus Scale (HRFS). The scale consists of eight 7-point scale (1=strongly disagree, 7=strongly agree) items (5 promotion items and 3 prevention items). We replaced “health” with “mental health” in the items so that the items are more relevant to mental health. An example of promotion focus is “If I see a good opportunity to improve my mental health, I take advantage of it right away,” and an example of prevention focus is “When I implement a mental health behavior, it’s because I want to protect myself from getting sick.” The scores are calculated by averaging promotion items and prevention items. HRFS demonstrated an advantage in predictive validity within health behavior contexts over general regulatory focus scales [52,53]. It also has adequate internal consistency, test-retest reliability, convergent validity, and discriminant validity [80]. In our study, HRFS showed internal consistency of *α*=.887 in promotion focus and *α*=.698 in prevention focus.

#### Perceived Difficulty of Making Choices and Preference for Choices

Perceived Difficulty of Making Choices (PDMC) and Preference for Choices (PFC) were measured with scales constructed and adopted by Feldman et al [48], which were positively associated with the Free Will and Determinism Scale [81]. Both scales consist of 2 items each with 7-point scales (1=strongly disagree, 7=strongly agree). An example item of PFC is “In each decision I face, I prefer to have as many options as possible to choose from,” and an example item of PDMC is “It’s very hard for me to choose between many alternatives.” Scale scores were calculated by averaging the items. The scales have not been formally validated. PFC scale shows an internal consistency of *α*=.785, and the internal consistency of PDMC is *α*=.690.

While we focused on preregistered confirmatory moderators in this manuscript, we included additional psychological attributes, notably Need For Cognition [82] in the same data collection, and the full list of these scales is reported in Multimedia Appendix 1 [31,52-67]. (We may conduct exploratory analyses with such variables for separate manuscripts and we welcome researchers to conduct secondary exploratory analyses [eg, machine learning methods, see discussion] with our open dataset.)

After completion of the above measures, participants were randomized to either (1) 1-Choice condition, (2) 4-Choice condition, or (3) 16-Choice condition. Each condition consists of an array of mental health self-care exercises with brief descriptions about the tasks involved and the purpose of each exercise. All exercises have the same purpose in improving mental well-being and reducing psychological distress and last for a few minutes. They are commonly used exercises in mindfulness-based, self-compassion-based, and cognitive-behavioral interventions, which are evidence-based approaches demonstrated to improve mental well-being and reduce anxiety and depressive symptoms [83-85]. In a previous randomized controlled trial conducted among Chinese in Hong Kong [84], brief mobile app-based mindfulness-based, self-compassion-based, and cognitive-behavioral interventions were found to be equivalent and effective in improving mental well-being and reducing psychological distress.

Half of the exercises involve writing tasks, and half of the exercises are experiential, based on audio/video. Examples include mindful awareness of emotions, mindful breathing (mindfulness), self-compassion letter-writing, self-kindness meditation (self-compassion), problem-solving, and absorbing activities (cognitive-behavioral). The order of the exercises was randomized to prevent order effects, and the choices of the single-option and 4-choice option (2 writing, 2 experiential) were randomized from the 16 choices. More details regarding the exercises and each condition are reported in the Multimedia Appendix 1 [31,52-67] “Details regarding Different Choice Sets and Exercises” section, with screenshots on Qualtrics, titles, and descriptions of all exercises presented or randomized. The Qualtrics QSF file is shared on OSF [86], in which people can download and upload the file on Qualtrics to view the experiment with exercises in the Chinese language.

After choosing “choose later,” “do not want to practice,” or choosing and involving in one of the exercises, participants were presented with the following variables. Variables designated as primary are variables for which we have specific hypotheses, and secondary are variables that are exploratory, as indicated in our preregistration.

#### Manipulation Check

We included a manipulation check: “The previous page has many mental health exercise choices,” with a 7-point scale (1=Strongly Disagree, 7=Strongly Agree), to test if the choice set size manipulation results in perceived differences in the number of choices across conditions. This question was presented neutrally to avoid negative or positive connotation regarding many choices, as there may be an impact on subsequent responses to the dependent variables if such a question was framed positively (diverse choices) or negatively (too many choices).

#### Decision Satisfaction (Primary)

Participants completed 2 items on decision satisfaction, which were based on Benoit and Miller’s [54,83] items. They included one item on satisfaction with the number of options and one item on satisfaction with decision experiences (the process). An example of a decision satisfaction item is “Are you satisfied or unsatisfied with the number of options just a moment ago?” Both items have 7-point scales (1=very unsatisfied, 7=very satisfied), which is the same as Benoit and Miller [54]. The internal consistency of the scale is *α*=.721.

#### Chosen Exercise Satisfaction (Primary)

Two items from Benoit and Miller [54,83] were used to measure chosen option satisfaction, including one item on the degree to which participants are satisfied or unsatisfied with the chosen exercise and one item on the degree to which participant believes the chosen exercise fulfills personal preferences. An example item is “Are you satisfied or unsatisfied with your chosen exercise just a moment ago?” The 2 items have 7-point scales (1=very unsatisfied, 7=very satisfied; 1=completely unable to fulfill my preference, 7=strongly fulfill my preference). The scale had an internal consistency of *α*=.763 in this study.

#### Completion (Primary)

Participants were asked whether they completed the exercise with a single question (“Did you complete the exercise just a moment ago?”) with three options: (1) did not choose any of the exercises, (2) completed, and (3) not completed. Only data from participants who chose one of the exercises were analyzed.

#### Engagement (Primary)

A single question on the degree of engagement in the exercise (“What was your level of engagement in the exercise just a moment ago?”) was used, with a 7-point scale (1=not engaged at all, 7=very engaged). Only data from participants who selected one of the exercises were analyzed.

We also included the following secondary measures for exploratory purposes. We did not have specific hypotheses as we are unaware of any study in the number-of-choice literature with such dependent variables, and at the preregistration stage, we perceived such variables as more distal compared with the primary measures we included. Despite that, we believed including these items would be worthwhile for several reasons. These measures are simple and not lengthy, particularly relevant in mental health contexts, and may inform future studies in the number-of-choice literature.

#### Attitudes Toward Chosen Exercise (Secondary)

Three items measuring participants’ attitudes toward chosen exercise were used, with one item each on liking, perceived helpfulness of the exercise, and desirability of the exercise. All these items are measured with 7-point scales (1=not desirable at all for me, 7=very desirable for me; 1=strongly dislike, 7=strongly like; 1=not helpful at all, 7=very helpful). An example item is “Do you think that the exercise you just did a moment ago was helpful for you?” The internal consistency was *α*=.903.

#### Perceived Improvement in Psychological State (Secondary)

Participants who chose one of the exercises also completed an item regarding their perceived improvement in mental state (“Do you think your mental state has improved or not due to the exercise a moment ago?). This item is measured with a 7-point scale (1=no improvement at all, 7=very substantial improvement).

Apart from the above secondary variables, 2 additional secondary items on behavioral intention in practicing the exercise in the future and engaging in our digital mental health platform were included. In retrospect, we consider these items to be too distal or not well-designed. These items are included in the shared datasets (Table S1 in Multimedia Appendix 1 [31,52-67] contains links), and other researchers may conduct analyses with such variables, while understanding the limitations of such variables.

After the first survey, participants were sent a link in which they could optionally click on to practice or not, with the same number and combination of options as the first survey. Two weeks later, they fill in questions regarding their experiences with similar measures as the first survey. As very few (lower than 15%) of participants engaged in practice after the first survey, and most of the items require participants to have chosen or practiced exercises after the first survey to be valid, a very low number of responses received in the follow-up survey (<100 participants) can be analyzed. As a result, our analyses in the results section focus on participants’ practice decisions, completion, and experiences of decision making and practices in the first survey, and the potential moderation effects.

### Ethical Considerations

We obtained ethics approval from the Chinese University of Hong Kong Survey and Behavioral Research Ethics Committee (number: SBRE-23‐0081). Participants provided informed consent via the web before the experiment, and the consent form indicated that they could withdraw from the experiment at any time. We emphasized in the consent form that participants would get compensated for their time regardless of whether they engaged in self-care exercise or not. Participants did not need to provide their names, and we noted in the consent form that their data would be confidential, anonymized, and deidentified. These practices may reduce social desirability bias in consumer research [87,88]. The datasets shared on OSF are anonymized and deidentified. Each participant was compensated with $50 Hong Kong dollars (equivalent to US $6.42) for completing the study, as transparently stated in the consent form.

## Results

### Notes Regarding Validation Check and Reported Results

We only reported results of participants who passed at least 1 validity check in the main manuscript. This procedure was not preregistered, but we believe this would be necessary to ensure the quality of the analyzed responses. For transparency purposes, we report results of all participants who were randomized to one of the conditions and completed one of the dependent variables in Multimedia Appendix 1 [31,52-67] (including participants who failed both attention checks). The results are highly similar.

### Manipulation Check

A one-way Welch ANOVA was conducted to test whether the manipulation was successful, in other words, whether there were significant differences in levels of agreement on the presence of many choices between participants in different conditions. Welch’s ANOVA was adopted instead of Fisher’s ANOVA as it is robust against unequal variances [89]. Results indicate that the manipulation was successful with substantial differences in perceived number of choices between conditions and are reported in the Manipulation Check Results section in Multimedia Appendix 1 [31,52-67].

### Alpha Adjustment for Multiple Testing

#### Alpha Adjustment Methods

As preregistered, to address risks of false positive results due to testing with multiple moderators and multiple dependent variables, the Holm sequential alpha adjustment method [55] was adopted for tests associated with our confirmatory hypotheses (with primary outcome variables). With the Holm method, the *P* values for each family were ranked from smallest to largest. For the lowest *P* value in the family, the alpha is adjusted by dividing 0.05 by the total number of tests in the family (0.05/m). For the second lowest *P* value in the family, the alpha is calculated by dividing 0.05 by m-1. For the third lowest *P* value, then adjusted alpha=.05/m^−2^, etc. As preregistered, the tests were divided into 2 families, each with different research objectives [90]. In Family A, tests were divided in relation to anxiety and depressive symptoms as well as state emotions (potential moderations of GAD-9, PHQ-7, and state emotions, with primary outcome variables listed above). In Family B, tests were divided in relation to potential moderation of chronic traits in motivation and decision making (HRFS, PDMC, and PFC).

Regarding our secondary (exploratory) dependent variables, we did not preregister any alpha adjustment method. For exploratory analyses, two approaches are common: (1) not adopting alpha adjustment [90-92], (2) adjusting alpha with less conservative and more powerful approaches such as Benjamini-Hochberg False Discovery Rate method [93,94]. We contemplated both approaches and decided to adopt the Benjamini-Hochberg method due to the substantial number of tests involved. With the Benjamini-Hochberg procedure, *P* values are ranked from smallest (order of 1) to largest. The adjusted alpha is calculated by rank/number of tests * 0.05. To illustrate, when the number of exploratory tests in a family is 24, the adjusted alpha for the lowest *P* value would be 1/24 * 0.05=0.00208333333, whereas the adjusted alpha for the 5th lowest *P* value would be 5/24 * 0.05=0.01041666666. Similarly, such tests were classified into families [90]: (1) comparison between different numbers of choices (main effect), (2) potential moderation of anxiety and depressive symptoms and state emotions, (3) potential moderation of psychological attributes.

#### Practice Exercise Decision

Using Jamovi [95], we conducted a logistic regression to compare participants’ likelihood of practicing (in the first survey) between 3 conditions. The chi-square goodness-of-fit test indicates nonequal distribution in practice decisions between conditions, *χ^2^*_2_=50.8, *P*<.001. Compared with the 1-choice condition (54/216, 24.8%), participants in the 4-choice condition (113/220, 50.7%) were substantially more likely to practice (ie, choose an exercise option), odds ratio (OR) 3.12, 95% CI (2.08-4.67), *P*<.001 (below adjusted alpha of .00416666666). Similarly, participants in the 16-choice condition (121/216, 55.8%) were much more likely to choose an exercise option, OR 3.83, 95% CI (2.55-5.76), *P*<.001 (below adjusted alpha of .00208333333), compared with participants in the 1-choice condition (54/216, 24.8%). However, we found no significant differences in practice likelihood between participants in the 16-choice condition (121/216, 55.8%) and the 4-choice condition (113/220, 50.7%), OR 1.23, 95% CI (0.84-1.79), *P*=.29.

#### Completion of Exercise

Another logistic regression was conducted to compare participants’ likelihood of completing chosen exercises between conditions. We found no support for differences in distributions of completion between conditions, *χ^2^*_2_=1.08, *P*=.583. The completion rates between conditions were not significantly different, 16-choice (106/139, 76.3%) versus 1-choice (90/112, 80.4%): OR 0.79, 95% CI (0.43-1.44), *P*=.44; 4-choice (111/137, 81.0%) versus 1-choice (90/112, 80.4%): OR 1.04, 95% CI (0.56-1.96), *P*=.90; 4-choice (111/137, 81.0%) versus 16-choice (106/139, 76.3%): OR 1.33, 95% CI (0.75-2.37), *P*=.34.

#### Decision Satisfaction

One-way Welch ANOVA was conducted, and differences in decision satisfaction between conditions were found, *F*_2, 432.00_=7.42, *P*<.001. Games-Howell *t*-tests were subsequently conducted. Participants in the 16-choice condition (n=216, mean 4.80, SD 1.13) had higher decision satisfaction compared with participants in the 1-choice condition (n=216, mean 4.41, SD 1.05), *t*_427.85_=3.77, *P*<.001 (below adjusted alpha of .00625), *d*=0.36, 95% CI (0.17-0.56). Tentative evidence indicates that participants in the 4-choice condition (n=220, mean 4.66, SD 1.06) have higher decision satisfaction than participants in the 1-choice condition, *t*_433.91_=2.52, *P*=.03 (above adjusted alpha of .00833333333), *d*=0.24, 95% CI (0.05-0.43). The difference between the 16-choice condition and the 4-choice condition did not reach significance, *t*_430.87_=1.36, *P*=.37, *d*=0.13, 95% CI (−0.06 to 0.32).

#### Other Immediately Measured Variables

Results from Welch’s ANOVAs showed no evidence for significant differences in other dependent variables, including chosen exercise satisfaction, engagement, attitude toward chosen exercise, and perceived improvement in mental state between conditions with different numbers of choices. Results are reported in Table 1.

**Table 1.** ANOVAs for chosen exercise satisfaction, exercise engagement, attitude toward chosen exercise, and perceived improvement in mental state.

Dependent variables	*F* test (*df*), *P* value	16-Choice versus 1-Choice Games-Howell *t* tests (*df*), *P* value	4-Choice versus 1-Choice Games-Howell *t* tests (df)*, P* value	16-Choice versus 4-Choice Games-Howell *t* tests (*df*), *P* value
Chosen exercise satisfaction	1.52 (2, 285.75); *P*=.22	1.74 (277.78); *P*=.19	0.85 (274.24); *P*=.67	0.93 (305.82); *P*=.62
Exercise engagement	0.98 (2, 246.61); *P*=.38	−1.00 (231.85); *P*=.58	−1.35 (237.95); *P*=.37	0.42 (268.80); *P*=.91
Attitude toward chosen exercise	0.34 (2, 243.07); *P*=.71	0.49 (228.05); *P*=.88	−0.28 (226.13); *P*=.96	0.82 (270.46); *P*=.69
Perceived improvement in mental state due to exercise	0.77 (2, 243.16); *P*=.46	1.04 (230.87); *P*=.55	0.01 (225.33); *P*>.99	1.09 (270.00); *P*=.52

#### Number of Practices After the First Survey

Furthermore, we found no evidence for differences in the number of practices (after the first survey) between participants in different conditions, *F*_2, 410.19_=0.13, *P*=.88. In other words, the differences in the number of practices after the first survey were not significantly different, 16-choice versus 1-choice, *t*_330.39_=0.50, *P*=.87; 4-choice versus 1-choice, *t*_391.77_=0.18, *P*=.98; 16-choice versus 4-choice, *t*_410.70_=0.32, *P*=.95.

### Moderation Analyses of Anxiety and Depressive Symptoms as Well as State Emotions

The following analyses were conducted with RStudio linear regression (lm) function [96].

### Linear Regressions With Anxiety Symptoms as the Potential Moderator

Findings from linear multiple regressions with anxiety symptoms (GAD-7) as the moderator found no support for significant moderation for decision satisfaction and exercise engagement. For chosen exercise satisfaction, attitude toward chosen exercise, and perceived improvement in mental state, we found tentative evidence for moderation. Results are summarized in Table 2. Simple slope analyses for chosen exercise satisfaction, attitude toward chosen exercise, and perceived improvement in mental state were conducted. A tentative moderating effect (with *P* values below .05 but above adjusted alpha) was found. People who experienced higher levels of anxiety symptoms, such that these participants in the 16-choice condition tended to experience higher chosen exercise satisfaction, more positive attitudes toward chosen exercise, and more perceived improvement in mental state due to chosen exercise, compared with corresponding participants in the 4-choice and 1-choice conditions (Table 3).

**Table 2.** Interaction results (linear regressions) with anxiety symptoms as the potential moderator.

Dependent variables	β (95% CI)	*P* value
Decision satisfaction	16-Choice versus 1-Choice * GAD:^a^ *β*=–.18 (–0.37 to 0.01) 16-Choice versus 4-Choice * GAD: *β*=–.11 (–0.30 to 0.08)	16-Choice versus 1-Choice * GAD: *P*=.06 16-Choice versus 4-Choice * GAD: *P*=.25
Chosen exercise satisfaction	16-Choice versus 1-Choice * GAD: *β*=–.30 (–0.53 to –0.08) 16-Choice versus 4-Choice * GAD: *β*=−.25 (–0.47 to –0.03)	16-Choice versus 1-Choice * GAD: *P*=.009 (above adjusted alpha of .00142857142) 16-Choice versus 4-Choice * GAD: *P*=.02 (above adjusted alpha of .00151515151)
Exercise engagement	16-Choice versus 1-Choice * GAD: *β*=−.08 (–0.34 to 0.18) 16-Choice versus 4-Choice * GAD: *β*=−.03 (–0.28 to 0.21)	16-Choice versus 1-Choice * GAD: *P*=.55 16-Choice versus 4-Choice * GAD: *P*=.78
Attitudes toward chosen exercise	16-Choice versus 1-Choice * GAD: *β*=−.33 (–0.59 to –0.08) 16-Choice versus 4-Choice * GAD: *β*=−.29 (–0.53 to –0.05)	16-Choice versus 1-Choice * GAD: *P*=.01 (above adjusted alpha of .00714285714) 16-Choice versus 4-Choice * GAD: *P*=.02 (above adjusted alpha of .01428571428)
Perceived improvement in mental state	16-Choice versus 1-Choice * GAD: *β*=−.36 (–0.61 to –0.10) 16-Choice versus 4-Choice * GAD: *β*=−.29 (–0.52 to –0.05)	16-Choice versus 1-Choice * GAD: *P*=.006 (above adjusted alpha of .00178571428) 16-Choice versus 4-Choice * GAD: *P*=.02 (above adjusted alpha of .01785714285)

a GAD: Generalized Anxiety Disorder.

**Table 3.** Simple slope analyses with Generalized Anxiety Disorder-7 (GAD-7) as the potential moderator, chosen exercise satisfaction, attitudes toward chosen exercise, and perceived improvement in psychological state as dependent variables.

GAD-7 level	Chosen exercise satisfaction, β (95% CI)	Attitudes toward chosen exercise, β (95% CI)	Perceived improvement in mental state, β (95% CI)
−1SD (lower anxiety)	1-Choice versus 16-Choice: *β*=.12 (–0.22 to 0.45); *P*=.49 4-Choice versus 16-Choice: *β*=.15 (–0.17 to 0.46); *P*=.36	1-Choice versus 16-Choice: *β*=.28 (–0.08 to 0.65); *P*=.13 4-Choice versus 16-Choice: *β*=.19 (–0.14 to 0.53); *P*=.26	1-Choice versus 16-Choice: *β*=.23 (–0.13 to 0.60); *P*=.21 4-Choice versus 16-Choice: *β*=.15 (–0.18 to 0.49); *P*=.38
Mean	1-Choice versus 16-Choice: *β*=−.20 (–0.43 to 0.04); *P*=.10 4-Choice versus 16-Choice: *β*=−.11 (–0.34 to 0.11); *P*=.31	1-Choice versus 16-Choice: *β*=−.06 (–0.31 to 0.19); *P*=.66 4-Choice versus 16-Choice: *β*=−.10 (–0.34 to 0.13); *P*=.39	1-Choice versus 16-Choice: *β*=−.13 (–0.38 to 0.12); *P*=.31 4-Choice versus 16-Choice: *β*=−.14 (–0.37 to 0.10); *P*=.26
+1SD (higher anxiety)	1-Choice versus 16-Choice: *β*=−.51 (–0.84 to –0.18); *P*=.002 (above adjusted alpha of .00138888888) 4-Choice versus 16-Choice: *β*=−.38 (–0.69 to –0.06); *P*=.02 (above adjusted alpha of .00147058823)	1-Choice versus 16-Choice: *β*=−.39 (–0.75 to –0.04); *P*=.03 (above adjusted alpha of 0.025) 4-Choice versus 16-Choice: *β*=−.40 (–0.74 to –0.06); *P*=.022 (above adjusted alpha of .02142857142)	1-Choice versus 16-Choice: *β*=−.49 (–0.85 to –0.13); *P*=.007 (above adjusted alpha of .00357142857) 4-Choice versus 16-Choice: *β*=−.43 (–0.76 to –0.09); *P*=.01 (above adjusted alpha of .00892857142)

#### Logistic Regressions With Anxiety Symptoms as the Potential Moderator, Practice Decision, and Completion as Dependent Variables

Logistic regressions were conducted to test the moderation of anxiety symptoms (GAD-7) in the impact of different numbers of choices on practice decision and completion. With practice decision as the dependent variable, we found no evidence for moderation, OR (GAD-7 * 4-choice versus 16-choice)=0.88 (95% CI 0.59-1.30), *P*=.52; OR (GAD-7 * 1-choice versus 16-choice)=0.91 (95% CI 0.60-1.38), *P*=.65. With completion as the dependent variable, we found no support for moderation, OR (GAD-7 * 4-choice versus 16-choice)=0.97 (95% CI 0.55-1.72), *P*=.93; OR (GAD-7 * 1-choice versus 16-choice)=0.98 (95% CI 0.54-1.79), *P*=.96.

#### Linear Regressions With Depressive Symptoms as the Potential Moderator

No support of depressive symptoms as moderation was found for various dependent variables, except attitudes toward chosen exercise. The interaction results are summarized in Table 4.

**Table 4.** Interaction results (linear regressions) with depressive symptoms as the potential moderator.

Dependent variables	β (95% CI)	*P* value
Decision satisfaction	16-Choice versus 1-Choice * PHQ:^a^ *β*=−.07 (–0.26 to 0.13) 16-Choice versus 4-Choice * PHQ: *β*=.01 (–0.18 to 0.20)	16-Choice versus 1-Choice * PHQ: *P*=.51 16-Choice versus 4-Choice * PHQ: *P*=.91
Chosen exercise satisfaction	16-Choice versus 1-Choice * PHQ: *β*=−.19 (–0.42 to 0.05) 16-Choice versus 4-Choice * PHQ: *β*=−.19 (–0.42 to 0.04)	16-Choice versus 1-Choice * PHQ: *P*=.12 16-Choice versus 4-Choice * PHQ: *P*=.11
Exercise engagement	16-Choice versus 1-Choice * PHQ: *β*=−.03 (–0.30 to 0.23) 16-Choice versus 4-Choice * PHQ: *β*=−.05 (–0.30 to 0.20)	16-Choice versus 1-Choice * PHQ: *P*=.80 16-Choice versus 4-Choice * PHQ: *P*=.69
Attitudes toward chosen exercise	16-Choice versus 1-Choice * PHQ: *β*=−.18 (–0.44 to 0.08) 16-Choice versus 4-Choice * PHQ: *β*=−.25 (–0.50 to –0.01)	16-Choice versus 1-Choice * PHQ: *P*=.18 16-Choice versus 4-Choice * PHQ: *P*=.04 (above adjusted alpha of .02857142857)
Perceived improvement in psychological state	16-Choice versus 1-Choice * PHQ: *β*=−.25 (–0.51 to 0.01) 16-Choice versus 4-Choice * PHQ: *β*=−.23 (–0.48 to 0.01)	16-Choice versus 1-Choice * PHQ: *P*=.07 16-Choice versus 4-Choice * PHQ: *P*=.06

a PHQ: Patient Health Questionnaire.

Specifically, the moderating effect of depressive symptoms on the number of choices (16-choice versus 4-choice) on attitudes toward exercise was inconclusive, with the *P* value below .05 (*P*=.04) but above the adjusted alpha based on Benjamini–Hochberg correction. Simple slope analysis using the PROCESS Macro function in R by Hayes [97] with +1SD value in depressive symptoms (PHQ=13.49) showed that participants in the 16-choice condition (mean=4.71) had more positive attitudes toward exercise compared with participants in the 4-choice condition (mean=4.27), *β*=.37 (95% CI 0.01-0.72), *P*=.04 (above adjusted alpha of .02678571428). Other regression coefficients at different levels of depressive symptoms were not significant.

#### Logistic Regressions With Depressive Symptoms as the Potential Moderator, Practice Decision and Completion as Dependent Variables

For practice decision, we found no support for interaction between depressive symptoms and number of choices, PHQ-9 * 4-choice versus 16-choice: OR 1.10 (95% CI 0.75-1.61), *P*=.63; PHQ-9 * 1-choice versus 16-choice: OR 1.32 (95% CI 0.87-2.01), *P*=.19. Additionally, we found no evidence for interaction between PHQ-9 and number of choices on completion of exercises, PHQ-9 * 4-choice versus 16-choice: OR 1.16 (95% CI 0.65-2.07), *P*=.62; PHQ-9 * 1-choice versus 16-choice: OR 1.35 (95% CI 0.73-2.50), *P*=.35.

#### Linear Regressions With State Emotions as the Potential Moderator

For decision satisfaction and engagement, no evidence for the moderation of state emotions was found. For chosen exercise satisfaction (primary dependent variable), we found conclusive evidence for the moderation of state emotions (*P*<.001, below adjusted alpha) when comparing the 16-choice condition with the 1-choice condition and found tentative evidence for the moderation of state emotions (*P*=.04) when comparing the 16-choice condition with the 4-choice condition. For secondary dependent variables, including attitudes toward chosen exercise and perceived improvement in psychological state, we found tentative evidence for the moderation of state emotions, and such results are reported in Table 5. Building on the moderation findings, simple slopes analyses were conducted. For people who are experiencing more intense unpleasant emotions, participants in the 16-choice condition tend to experience higher chosen exercise satisfaction compared with participants in the 1-choice condition, whereas there is tentative evidence of higher chosen exercise satisfaction for people in the 16-choice condition compared with participants in the 4-choice condition. Furthermore, we found support that participants who experienced more intense unpleasant emotions in the 16-choice condition experienced more positive attitudes toward the chosen exercise, compared with participants in the 4-choice condition, and tentative evidence for differences in chosen exercise satisfaction between the 16-choice condition and the 1-choice condition. Moreover, we found tentative evidence that people with more intense unpleasant emotions in the 16-choice condition experienced more subjective improvement in mental state compared with participants in the 1-choice condition. We report more details of simple slope analyses in Tables6  7.

**Table 5.** Interaction results (linear regressions) with state emotions (continuous) as the potential moderator.

Dependent variables	β (95% CI)	*P* value
Decision satisfaction	16-Choice versus 1-Choice * State Emotions: *β*=.17 (–0.01 to 0.36) 16-Choice versus 4-Choice * State Emotions: *β*=.01 (–0.17 to 0.20)	16-Choice versus 1-Choice * State Emotions: *P*=.08 16-Choice versus 4-Choice * State Emotions: *P*=.88
Chosen exercise satisfaction	16-Choice versus 1-Choice * State Emotions: *β*=.41 (0.18 to 0.64) 16-Choice versus 4-Choice * State Emotions: *β*=.23 (0.01 to 0.45)	16-Choice versus 1-Choice * State Emotions: *P*<.001 (below adjusted alpha of .00135135135) 16-Choice versus 4-Choice * State Emotions: *P*=.04 (above adjusted alpha of .00161290322)
Exercise engagement	16-Choice versus 1-Choice * State Emotions: *β*=.18 (–0.08 to 0.44) 16-Choice versus 4-Choice * State Emotions: *β*=.14 (–0.11 to 0.38)	16-Choice versus 1-Choice * State Emotions: *P*=.19 16-Choice versus 4-Choice * State Emotions: *P*=.27
Attitudes toward chosen exercise	16-Choice versus 1-Choice * State Emotions: *β*=.36 (0.10 to 0.62) 16-Choice versus 4-Choice * State Emotions: *β*=.29 (0.05 to 0.53)	16-Choice versus 1-Choice * State Emotions: *P*=.007 (above adjusted alpha of .00535714285) 16-Choice versus 4-Choice * State Emotions: *P*=.02 (above adjusted alpha of .01607142857)
Perceived improvement in mental state	16-Choice versus 1-Choice * State Emotions: *β*=.32 (0.07 to 0.58) 16-Choice versus 4-Choice * State Emotions: *β*=.17 (–0.07 to 0.41)	16-Choice versus 1-Choice * State Emotions: *P*=.014 (above adjusted alpha of .01071428571) 16-Choice versus 4-Choice * State Emotions: *P*=.16

**Table 6.** Simple slope analyses with state emotions as the potential moderator, chosen exercise satisfaction, attitudes toward chosen exercise, and perceived improvement in mental state as dependent variables.

State emotions	Chosen exercise satisfaction, β (95% CI)	Attitudes toward chosen exercise, β (95% CI)	Perceived improvement in mental state, β (95% CI)
−1 SD (stronger unpleasant emotions)	1-Choice versus 16-Choice: *β*=−.60 (–0.92 to –0.28); *P*<.001 (below adjusted alpha of .00131578947) 4-Choice versus 16-Choice: *β*=−.35 (–0.67 to –0.03); *P*=.03 (above adjusted alpha of .0015625)	1-Choice versus 16-Choice: *β*=−.40 (–0.75 to –0.05); *P*=.03 (above adjusted alpha of .02321428571) 4-Choice versus 16-Choice: *β*=−.40 (–0.75 to –0.06); *P*=.02 (above adjusted alpha of .01964285714)	1-Choice versus 16-Choice: *β*=−.44 (–0.79 to –0.09); *P*=.02 (above adjusted alpha of .0125) 4-Choice versus 16-Choice: *β*=−.31 (–0.66 to 0.03); *P*=.07
Mean	1-Choice versus 16-Choice: *β*=−.18 (–0.41 to 0.05); *P*=.12 4-Choice versus 16-Choice: *β*=−.12 (–0.34 to 0.10); *P*=.30	1-Choice versus 16-Choice: *β*=−.04 (–0.29 to 0.21); *P*=.73 4-Choice versus 16-Choice: *β*=−.11 (–0.35 to 0.12); *P*=.36	1-Choice versus 16-Choice: *β*=−.11 (–0.37 to 0.14); *P*=.38 4-Choice versus 16-Choice: *β*=−.14 (–0.38 to 0.10); *P*=.25
+1 SD (stronger pleasant emotions)	1-Choice versus 16-Choice: *β*=.24 (–0.10 to 0.57); *P*=.17 4-Choice versus 16-Choice: *β*=.12 (–0.19 to –0.43); *P*=.46	1-Choice versus 16-Choice: *β*=.31 (–0.05 to 0.68); *P*=.10 4-Choice versus 16-Choice: *β*=.18 (–0.15 to 0.51); *P*=.28	1-Choice versus 16-Choice: *β*=.21 (–0.16 to 0.58); *P*=.26 4-Choice versus 16-Choice: *β*=.03 (–0.30 to 0.37); *P*=.84

**Table 7.** Means of 1-Choice, 4-Choice, and 16-Choice conditions at different levels of state emotions.

	1-Choice	4-Choice	16-Choice
−1 SD (stronger unpleasant emotions)	Chosen exercise satisfaction: 4.13 Attitude toward exercise: 4.22 Perceived Improvement in psychological state: 3.57	Chosen exercise satisfaction: 4.41 Attitude toward exercise: 4.21 Perceived improvement in psychological state: 3.75	Chosen exercise satisfaction: 4.80 Attitude toward exercise: 4.71 Perceived improvement in psychological state: 4.18
Mean	Chosen exercise satisfaction: 4.48 Attitude toward exercise: 4.50 Perceived improvement in psychological state: 3.91	Chosen exercise satisfaction: 4.56 Attitude toward exercise: 4.42 Perceived improvement in psychological state: 3.87	Chosen exercise satisfaction: 4.69 Attitude toward exercise: 4.55 Perceived improvement in psychological state: 4.07
+1 SD (stronger pleasant emotions)	Chosen exercise satisfaction: 4.84 Attitude toward exercise: 4.78 Perceived improvement in psychological state: 4.25	Chosen exercise satisfaction: 4.71 Attitude toward exercise: 4.62 Perceived improvement in psychological state: 4.00	Chosen exercise satisfaction: 4.57 Attitude toward exercise: 4.40 Perceived improvement in psychological state: 3.95

#### Logistic Regressions With State Emotions as the Potential Moderator

With practice decision as the dependent variable, the regression indicates no support for moderation, OR (State Emotions * 4-choice vs 16-choice)=1.03 (95% CI 0.70-1.50), *P*=.89; OR (State Emotions * 1-choice vs 16-choice)=0.92 (95% CI 0.61-1.40), *P*=.71. With completion as the variable, we also found no evidence for moderation, OR (State Emotions * 4-choice vs 16-choice)=0.85 (95% CI 0.47-1.53), *P*=.59; OR (State Emotions * 1-choice vs 16-choice)=1.18 (95% CI 0.64-2.16), *P*=.60.

### Moderation Analyses of Individual Characteristics

#### Linear Regressions With Health Regulatory Focus [80] as the Potential Moderator

Interaction results of health regulatory focus with different dependent variables were all nonsignificant and are summarized in Table S3 in Multimedia Appendix 1 [31,52-67].

#### Logistic Regressions With Health Regulatory Focus [80] as the Potential Moderator With Practice Decision and Completion as Dependent Variables

With logistic regression, we found no evidence for moderation of health regulatory focus on the impact of number of choices in practice decision, OR (HRFS * 4-choice vs 16-choice)=1.01 (95% CI 0.70-1.47), *P*=.95; OR (HRFS * 1-choice vs 16-choice)=0.77 (95% CI 0.50-1.20), *P*=.26. With completion as the dependent variable, we also found no support for moderation of health regulatory focus, OR (HRFS * 4-choice vs 16-choice)=0.94 (95% CI 0.51-1.72), *P*=.84; OR (HRFS * 1-choice vs 16-choice)=0.67 (95% CI 0.35-1.28), *P*=.22.

#### Linear Regressions With PFC and PDMC [48] as the Potential Moderator

With PFC as the potential moderator, we found no support for any interaction effects with all continuous variables. Results are summarized in Table S4 in Multimedia Appendix 1 [31,52-67]. Additionally, we found no evidence for moderation with PDMC with all continuous variables (Table S5 in Multimedia Appendix 1 [31,52-67]).

#### Logistic Regressions With PFC and PDMC [48] as the Potential Moderator

With practice decision as the dependent variable, we found no evidence for moderation with PDMC as the potential moderator, OR (PDMC * 4-choice vs 16-choice)=0.86 (95% CI 0.58-1.27), *P*=.45; OR (PDMC * 1-choice vs 16-choice)=1.23 (95% CI 0.82-1.85), *P*=.31. With completion as the dependent variable and PDMC as the potential moderator, the results also indicate no significant moderation, OR (PDMC * 4-choice vs 16-choice)=1.05 (95% CI 0.57-1.92), *P*=.88; OR (PDMC * 1-choice vs 16-choice)=0.98 (95% CI 0.53-1.79), *P*=.95. Additionally, we found no support for moderation of preferences for choices with practice decision as the dependent variable, OR (PFC * 4-choice vs 16-choice)=0.98 (95% CI 0.66-1.44), *P*=.91; *OR* (PFC * 1-choice vs 16-choice)=0.79 (95% CI 0.52-1.20), *P*=.27. Finally, we found no evidence for moderation of PFC with completion of exercise as the dependent variable, OR (PFC * 4-choice vs 16-choice)=0.92 (95% CI 0.50-1.67), *P*=.78; OR (PFC * 1-choice vs 16-choice)=1.02 (95% CI 0.56-1.84), *P*=.96.

## Discussion

### Principal Results: Comparing Different Numbers of Choices (Overall Effects)

Our investigation delved into potential differences in various outcomes across different numbers of choices. Participants in 4-choice and 16-choice conditions appeared more likely to practice one of the mental health self-care exercises and reported higher levels of decision satisfaction, compared with participants in the single-choice condition. These findings align with existing studies that underscore the positive impact of providing multiple choices, fostering increased active involvement and satisfaction among participants in health interventions [16].

The rationale behind these results might be elucidated using the self-determination theory. Individuals might be more inclined to engage in mental health behaviors when granted autonomy to make a choice when given multiple options [5,13-15]. The provision of multiple options caters to the fundamental human need for autonomy and the freedom to choose, possibly leading to heightened decision satisfaction in the multi-choice conditions compared with the single-choice condition. Conversely, individuals presented with only one option, including Chinese people, may experience a sense of choice deprivation and feel less satisfied with the insufficient number of choices [98].

Another plausible explanation rooted in classical economic models suggests that in conditions offering multiple choices, participants are more likely to discover an exercise aligning with their preferences, thus increasing the likelihood of selection [3,4,18]. In contrast, the singular exercise choice provided may not resonate with participants’ preferences, leading to lower exercise selection rates. However, our findings found no support for significant differences between the multiple-choice conditions and the single-choice condition in other outcomes, such as satisfaction with the chosen exercise, engagement levels, and completion rates. This lack of differentiation could stem from these variables predominantly reflecting experiences with the specific chosen exercise, with the benefits of multiple-choice provision potentially not extending to these more distal variables.

While our study revealed distinctions in 2 outcome variables between the single-choice and 4-choice conditions, as well as between the single-choice and 16-choice conditions, in terms of overall effects, we did not observe any significant differences in dependent variables between the 4-choice and 16-choice conditions. These findings align with the meta-analysis on choice set size conducted by Scheibehenne et al [30], which reported a negligible mean effect size when comparing higher versus lower numbers of choices. Similarly, our results are consistent with the meta-analysis by Chernev et al [4], which found no substantial differences in outcomes, including satisfaction and option selection, when comparing larger and smaller choice sets, among studies examining potential moderators. The average effect sizes of main effects, particularly in studies testing potential moderators, often tend to be minimal or statistically nonsignificant [4]. While some studies find support for the overchoice effect (eg, [26]), others (eg, [99]) found better outcomes with a higher number of choices, underscoring the variability of effects across different contexts [4].

The reasons behind minimal differences between the 16-choice condition and the 4-choice condition remain unclear, and our findings contradict concerns by some digital mental health researchers who suggested a higher number of choices may backfire [1,42]. It is possible that discomfort or indecisiveness (not selecting any of the choices) due to many-choice comparison is less likely to occur in decision-making contexts where making a choice does not involve any financial costs, including the context of our experiment. However, it is unclear if our findings are generalizable to digital mental health platforms that require payments for audio, video, and written exercises. In contexts where people need to pay the prices, the overchoice effect may be more likely to occur, possibly due to counterfactual thinking, anticipated and experienced regret, or other uncomfortable emotions regarding the monetary costs [4,11,26,100-102]. That said, we are unaware of studies that compare the impact of choice set sizes in decision-making contexts with and without monetary considerations. These explanations are speculative, and further research, comparing digital health self-care choices that do not require payment versus self-care choices that require payment, is warranted to delve deeper into these nuances and test these conjectures.

### Principal Results: Tentative Evidence for Moderations of State Emotions and Anxiety

Contrary to our hypotheses, recent findings in the broader overchoice literature beyond mental health contexts [32], and concerns raised by digital mental health researchers [1,42], our study unearthed tentative evidence suggesting that individuals with heightened anxiety levels and stronger negative emotional states may actually experience greater satisfaction, exhibit more positive attitudes toward the selected exercise, and perceive greater psychological improvement when presented with a higher number of choices (16 choices), as opposed to those offered a single choice or 4 choices. Our expectations were rooted in the notion that individuals experiencing intense anxiety and unpleasant emotions might struggle with decision-making in larger choice sets due to limited cognitive resources and heightened difficulties, leading to unpleasant experiences when choosing from a wider array of options [1,32,38,39,41]. However, our findings diverged from this anticipated direction, prompting a reflection. How can we interpret these unexpected results, and are these results reliable?

Our moderation findings are tentative, but we believe such results are likely reliable despite some uncertainties. While for several dependent variables, our results (moderation of anxiety symptoms and state emotions) reached conventional statistical significance of *P*<.05, most of the results did not retain significance following alpha corrections. There exists a possibility that these findings could be attributed to chance, particularly considering the inclusion of multiple potential moderators and a range of primary and secondary dependent variables. Despite this uncertainty, the likelihood of these results being false positives is relatively low, given the medium to large effect sizes observed for the aforementioned dependent variables, based on the benchmark outlined by Fey et al [103]. Notably, effect size serves as a robust indicator of the replicability of research findings [104]. Moreover, a common thread among the variables with *P*<.05 results is that they are all regarding participants’ subjective perceptions and sentiments (satisfaction, attitude, and perceived psychological improvement) concerning the selected exercise, rather than behavioral measures like practice decision and completion. Moderations of both anxiety symptoms and emotional states concerned these same subjective dependent variables, suggesting that moderations of anxiety and emotional states for such outcomes likely exist.

For participants with high anxiety and stronger unpleasant emotions, the higher satisfaction, more positive attitudes, and more subjective improvement in mental state with such exercises under a larger choice set may stem from a higher probability of matching their preferences and needs. This resonates with economic models and studies positing that individuals have a higher probability of finding options that cater to their specific preferences and goals when faced with more choices, as previously discussed [3,4,17]. Crucially, this could also be attributed to the heightened desire of individuals with elevated anxiety levels and unpleasant emotions to alleviate their uncomfortable feelings. A selection pool of 16 exercises is more likely to include self-care activities that effectively address their needs, in contrast to single or limited 4-choice sets. Individuals may perceive only a small fraction of exercises (perhaps one or a few) as beneficial for their personal well-being. When the choice set comprises only 1 or 4 exercises, the chances of encountering an emotion-relief method that precisely aligns with their individual needs are considerably lower. This disparity underscores the potential advantages of a larger choice set over a smaller choice set in matching personal needs, preferences, and goals for individuals experiencing high anxiety and unpleasant emotions.

Related to the heightened need to address uncomfortable emotions, when people experience unpleasant mental health and emotional states, mental health–related exercises may be perceived as more personally relevant. Based on dual process models such as the Elaboration Likelihood Model and Heuristic-Systematic Information Processing Theory, heightened perceived personal relevance can result in higher effort in information processing [105,106]. It is possible that such heightened effort in information processing and more elaborate processes in thinking and decision making may increase the likelihood of participants choosing exercises that can better satisfy their emotional needs and help address their distress, through considering more options effortfully and deciding systematically with a larger choice set. In contrast, smaller choice sets may not align well with such more elaborate and effortful processes.

Conversely, individuals with lower levels of anxiety and unpleasant emotions may not have any need to further regulate their mental health at the time of the experiment and thus may consider mental health exercises as less personally relevant. Consequently, the utility of a larger choice set may hold less significance or may be perceived as irrelevant to them. As such, they may expend less cognitive effort in processing information and weighing different options, which is consistent with dual process models discussed above. Given that practicing these self-care exercises may be considered less relevant to this group of participants, the number of choices presented may yield minimal, if any, discernible effects. This rationale may explain why we did not uncover substantial differences in both behavioral responses and subjective experiences among individuals with lower levels of anxiety and unpleasant emotions across varying choice set sizes.

It is hard to interpret the absence of evidence for the moderating effect of depressive symptoms, as null findings do not necessarily imply the absence of effect. One interpretation is that, as the direction of findings is consistent with anxiety and state emotions, moderation may exist but was not detected due to relatively small effect sizes. Therefore, the moderation effect of depressive symptoms was nonsignificant. Further research with a larger sample size and a wider range of anxiety and depressive symptoms is needed.

### Limitations, Strengths, and Future Research Directions

The above explanations regarding the moderations of anxiety symptoms and state emotions are speculative. The study did not delve into testing potential mediating mechanisms, and future research can explore factors such as perceived need to alleviate unpleasant emotions, perceived personal relevance of mental health self-care exercises, and elaboration in processing and deciding on mental health exercises. Through such mediated analyses, mechanisms of the phenomenon can be better understood. Additionally, it remains possible that the moderation findings can be better elucidated by a third variable linked to anxiety and unpleasant emotions, such as fear of missing out [107,108]. Subsequent studies can incorporate these variables to further investigate and validate these potential explanations.

Given the tentative nature of our moderation findings and the lack of significance in most results following alpha corrections, it is imperative to conduct replications and extensions to ascertain the replicability, reliability, and generalizability of the findings. These efforts can involve close or very close replications (directing repeating our work with similar/same designs), or conceptual replications [109] within the realm of digital mental health platforms or diverse contexts like other digital health arenas or consumer decision-making scenarios. Future research initiatives can aim to investigate whether these effects are specific to the digital mental health domain or if they hold broader generalizability and applicability across various decision-making contexts.

An intriguing avenue for conceptual replications-extensions, or follow-up studies, involves investigating the impact of recommendation statements when presenting varying choice sets (ie, a single choice, 4 choices, or 16 choices) on decision outcomes. For instance, investigating how phrases like “We strongly recommend you to practice the following exercise(s)” influence individuals’ choices and experiences compared with conditions without such recommendation language may yield valuable insights. Notably, our study did not incorporate recommendation wordings in any of the conditions, and it is plausible that the inclusion of such language would lead to increased engagement, decision-making, satisfaction, and other subjective outcome variables [1]. Introducing recommendation wordings in select conditions can also enhance the ecological validity of the results, considering that recommendation systems are increasingly common in digital mental health platforms [1,110]. Exploring the influence of recommendation statements within the context of different choice set sizes can provide a deeper understanding of how decision-making processes and subjective experiences are influenced in digital mental health settings and beyond.

Related to recommendations, another worthwhile direction is to compare different numbers of choices with and without tailored recommendations, which have been commonly adopted and researched in digital mental health contexts [1,110,111]. Studies can test if tailored recommendations that are based on prior behavioral data, demographics, mental health states, and other psychological attributes and offered a lower number of choices may outperform a higher number of choices that are not tailored, as such fewer number of choices may be able to align with service users’ needs and preferences better without providing a large amount of information. Studies that involve the intersection of choice set size and tailored recommendations [111] will require a much larger sample size due to a much higher number of conditions but can be feasible in digital mental health platforms where there are many service users. Another method that has been adopted in digital health studies and can be considered in testing the effects of various conditions over time is micro-randomized controlled trials, in which service users or participants are randomized sequentially and multiple times [112,113]. Further research can help us understand if more choices are really needed to achieve matching of needs, or if a lower number of choices of tailored recommendations can already satisfy users’ needs. It is possible that when comparing subjective experiences and behavioral outcomes across conditions, mental health states and psychological attributes (eg, trait reactance, [114]) may moderate such effects.

Another worthwhile direction in replications-extensions or follow-up studies is to investigate other choice set sizes. As mentioned in the introduction, the 3 choice set sizes were chosen as such sizes were potentially suitable for different subgroups of participants and feasible to implement in real-world mobile apps. Over 30 choices are perhaps overwhelming due to lower mental health literacy of most Chinese people [21,22], as there may be too much unfamiliar and technical information to process and understand (see studies by Chernev et al [4], Misuraca et al [5], and Mogilner et al [24] regarding higher likelihood of overchoice effect among people with lower familiarity or expertise with the options). However, it is possible that such a number of choices may be suitable for a proportion of participants with higher digital mental health self-care related knowledge, since studies in the broader choice set size literature have shown that people with higher expertise regarding the options benefit more from a larger choice set [4,5,24]. While such a choice set size of over 30 choices does not seem suitable in a mobile app, it can be feasible in a web page in a computer. Another possibility is that a number between 4 and 16 may outperform other numbers of choices for a proportion of participants, perhaps because a smaller choice set does not optimally satisfy needs and preferences, but a larger choice set is also suboptimal due to increased cognitive costs and diminishing marginal benefits, whereas a medium choice set may satisfy needs well with limited cognitive costs (see studies by Grant and Schwartz [115], Liu et al [116], and Reutskaja and Hogarth [117] regarding inverted-U relationship between number of choices and outcomes). Further digital health studies can examine this speculation of an inverted-U relationship. That said, including more conditions with different choice set sizes will require more participants, and these decisions involve dilemmas given considerations for power analysis, number of participants, and real-world constraints in data collection.

Another worthwhile area for improvement in future replications, extensions, and follow-up studies is to include a question on perceived quality to test if the perceived quality of exercises is similar or varies between conditions. Given that exercises in the 1-choice and 4-choice conditions were randomly chosen from the exercises that were also presented in the 16-choice condition, it is likely that perceived quality would not vary much. That said, perceived quality between conditions may influence the results, and future studies can explicitly account for this.

Furthermore, researchers may consider investigating decision-making dynamics, task completion rates, user experiences, and mental health outcomes over extended periods within a digital platform, where individuals repeatedly engage with self-care or health-related options. Conducting such longitudinal studies would necessitate larger sample sizes to ensure adequate statistical power, as sustained engagement in self-care exercises or digital interventions is not common among most individuals [118,119]. These studies can be effectively carried out in real-world digital platforms with a substantial user base, ensuring sufficient statistical power and enhancing ecological validity, while examining generalizability and applicability.

In addition to pursuing replications-extensions or follow-up studies involving primary data collection and analyses, another valuable direction for research involves conducting secondary data analyses with our datasets shared on the OSF (Table S1 in Multimedia Appendix 1 [31,52-67] contains links). Given the tentative nature of our findings, exploring the data with different statistical methods may lead to different findings, offer fresh perspectives, and potentially uncover new insights. Researchers can consider using methodologies like the Personalized Advantage Index [120] and machine learning techniques such as Random Forests to delve into potential moderators and enhance personalization strategies [56]. Moreover, while our manuscript primarily focused on preregistered confirmatory moderators and hypotheses, as well as a few exploratory outcome variables, we included additional measures of psychological attributes such as Need for Cognition [82] in our data collection (Multimedia Appendix 1 [31,52-67]). We welcome and encourage researchers to conduct secondary analyses based on our dataset and are open to potential collaborations in this regard.

### Implications

Presenting a higher number of choices does not appear to be counterproductive for most individuals with higher levels of anxiety symptoms, depressive symptoms, or intense state unpleasant emotions. For individuals experiencing elevated anxiety and depression symptoms or unpleasant emotions, there is currently no empirical evidence supporting the reduction of presented choices if there are initially 10‐20 options shown. Instead, our findings tentatively indicate that offering a greater number of choices (16 options) may actually enhance the subjective experiences of individuals with high anxiety and intense unpleasant emotions. This could stem from an increased likelihood of aligning with and addressing their mental health needs with a larger choice set. However, we cannot definitively ascertain the outcomes of presenting individuals with an even larger number of choices (eg, over 30 choices, see Chernev et al [4] meta-analysis), and caution is advised for digital mental health developers and designers when presenting dozens of options.

In the realm of digital mental health platforms, many already incorporate measures like GAD-7 for assessing anxiety, where our findings find support for moderations based on anxiety symptoms. Tailoring the number of choices to individual anxiety levels may be beneficial. Furthermore, our moderation results concerning state unpleasant emotions suggest that including measures of current emotional states may be valuable. Developers of digital mental health platforms also need to consider the impact of adding additional items, such as 8 new state emotion measures, to existing assessments, as this may deter some participants from completing them. One potential strategy could involve including 1 to 3 state emotion items that exhibit stronger or more consistent moderation effects compared with others. Digital mental health platforms may include multiple state emotion items for a period of time. After that, based on behavioral, satisfaction, and subjective experiences data and corresponding moderation analyses (eg, through machine learning methods such as Random Forest [56]) with the initially included emotions, they may choose 1 to 3 state emotions that show stronger moderation effects to increase the likelihood that people fill in such measures and to facilitate personalization for a higher proportion of participants.

### Conclusions

Our research found support for the advantage of having multiple exercise choices (4 choices or 16 choices) in enhancing the likelihood of practicing self-care and decision satisfaction, surpassing such outcomes of the single choice condition. More importantly, contrary to our expectations, prior findings [32], and concerns regarding the impact of a larger choice set in the digital mental health field [1,42], we found tentative evidence that for people with more anxiety symptoms and more intense unpleasant emotions, presenting them with a higher number of choices (16 choices) would be more beneficial for their subjective experiences of the chosen exercise (ie, satisfaction, attitude, perceived improvement in mental state) than presenting them with a lower number of choices (4 choices or 1 choice). This unexpected finding could be attributed to the higher probability of encountering exercises that align with participants’ mental health needs, preferences, and effectively address their emotional distress within a more extensive choice array.

In light of these intriguing findings, we advocate for further investigations into the underlying mechanisms at play, replication and extension studies to examine the reliability and generalizability of our findings, perhaps with additional choice set size conditions, secondary analyses using our open dataset, and additional research on the intersection between tailored recommendations and choice set size, as well as impact of choice set size in decision-making contexts without monetary considerations. Moreover, we highlight the potential of tailoring the number of choices based on individuals’ anxiety symptoms and current emotional states within digital mental health platforms. We call for further investigation aimed at deepening our understanding of how choice set sizes can be potentially tailored to enhance engagement, user experiences, and outcomes in digital mental health environments.

## Supplementary material

10.2196/71165 Multimedia Appendix 1 Supplementary file with OSF links, supplementary results, and details regarding method or materials.

## Abbreviations

GAD-7: Generalized Anxiety Disorder-7
HRFS: Health Regulatory Focus Scale
OR: odds ratio
OSF: Open Science Framework
PDMC: Perceived Difficulty of Making Choices
PFC: Preference for Choices
PHQ-9: Patient Health Questionnaire-9
