# Supplementary material for: Effects of Choice Set Sizes and Moderations of Anxiety and State Emotions on Mental Health Self-Care Uptake, Engagement, and User Experience: Experimental Study
Source: JMIR Hum Factors. 2025 Dec 15;12:e71165. doi: 10.2196/71165 (PMC12705060; doi:10.2196/71165)
Supplement: Multimedia Appendix 1 [file humanfactors-v12-e71165-s001.docx]

**Supplementary**

**Open Science Framework Files and Links**

The Open Science Framework (OSF) project page, with the files, is here: <https://osf.io/puwxb/>

**Table S1.** Filenames, Contents, and Links of Documents on OSF

| **Filename** | **Content** | **Link** |
| --- | --- | --- |
| Number of Choices Digital Mental Health Exercise Pre Registration Editted 501.docx | Pre-registration document | https://osf.io/yxc6q |
| Choice_Empowerment_Choice_Overload_Exercise_Study_16-Option_Condition.qsf | Qualtrics File for 16-Choice Condition Optional Self-Care Exercise (After the first survey) | https://osf.io/b7fhk |
| Choice_Empowerment_Choice_Overload_Exercise_Study_1-Option_Condition.qsf | Qualtrics File for 1-Choice Condition Optional Self-Care Exercise (After the first survey) | https://osf.io/spg2d |
| Choice_Empowerment_Choice_Overload_Exercise_Study_4-Option_Condition.qsf | Qualtrics File for 4-Choice Condition Optional Self-Care Exercise (After the first survey) | https://osf.io/5kcbz |
| Choice_Empowerment_Choice_Overload_Study_First_Survey_-_Revised.qsf | Qualtrics File for First Survey | https://osf.io/rgm25 |
| Choice_Empowerment_Choice_Overload_Study_Follow_Up_and_Debriefing.qsf | Qualtrics File For Final Survey | https://osf.io/3yqmj |
| Number of Choices Study Analysis.omv | Jamovi file for analyses after exclusion of invalid responses – focusing on internal consistency tests and logistic regressions | https://osf.io/pn6b3 |
| Number of Choices Study Analysis Pre Exclusion.omv | Jamovi file for analyses before exclusion of invalid responses | https://osf.io/y4pb3 |
| Number of Choice Study 16-Choice Condition Removing Personal Info.xlsx | Raw datafile of 16-Choice Condition Optional Self-Care Activities, with personally sensitive information removed (e.g. IP address, name, etc) | https://osf.io/bfzr8 |
| Number of Choice Study 1-Choice Condition Removing Personal Info.xlsx | Raw datafile of 16-Choice Condition Optional Self-Care Activities | https://osf.io/b9vyg |
| Number of Choice Study 4-Choice Condition Removing Personal Info.xlsx | Raw datafile of 16-Choice Condition Optional Self-Care Activities | https://osf.io/6zqwy |
| Number of Choice Study Follow Up Removing Personal Info.xlsx | Raw datafile of Follow-up Survey | https://osf.io/guzv2 |
| Number of Choice Study Removing Personal Info Pre Cleaning.xlsx | Raw datafile of First Survey | https://osf.io/d6c8a |
| Number of Choice Study.xlsx | Cleaned and combined datafile | https://osf.io/9w47m |
| Number of Choice Study Analyses Jan 2025.Rmd | RMarkdown code | https://osf.io/5nspz |
| Number-of-Choice-Study-Analyses-Jan-2025.html | RMarkdown knitted HTML | https://osf.io/s5qgf |

**Manipulation Check Results**

Significant differences between conditions were found, *F*(2, 427) = 73.3, *p* < .001. The Games-Howell t-tests revealed that 1) participants in the 16-choice condition had higher agreement that there are “many choices” compared to participants in the 4-choice condition, *t*(437) = 6.30, *p* < .001, *d* = 0.60, 95% CI [0.41, 0.79], 2) participants in the 16-choice condition agreed more strongly that there are “many choices” than participants in the 1-choice condition, *t*(398) = 12.05, *p* < .001, *d* = 1.21, 95% CI [0.99, 1.42], 3) participants in the 4-choice condition agreed more that there are “many choices” than those in the 1-choice condition *t*(396) = 6.83, *p* < .001, *d* = 0.69, 95% CI [0.48, 0.89]. Such results indicate the manipulation was successful. Descriptive statistics of levels of agreement of different conditions are reported in Table S2.

**Table S2.** Descriptive Statistics in Manipulation Check Question of 1-Choice, 4-Choice, and 16-Choice Conditions

| Condition | Number of Participants in the Condition | Mean | Standard Deviation |
| --- | --- | --- | --- |
| 1-Choice | 216 | 3.80 | 1.75 |
| 4-Choice | 223 | 4.80 | 1.30 |
| 16-Choice | 217 | 5.59 | 1.31 |

**Interaction Results for Psychological Attribute Variables as Potential Moderators**

**Table S3.** Interaction Results (Linear Regressions) with Health Regulatory Focus as the Potential Moderator

| Dependent Variables | *β* [95% CI] | *p* |
| --- | --- | --- |
| Decision Satisfaction | 16-Choice versus 1-Choice * HRFS^a^: *β* = 0.08 [-0.11, 0.28]  16-Choice versus 4-Choice * HRFS: *β* = 0.11 [-0.07, 0.30] | 16-Choice versus 1-Choice * HRFS: *p* = .398  16-Choice versus 4-Choice * HRFS: *p* = .207 |
| Chosen Exercise Satisfaction | 16-Choice versus 1-Choice * HRFS: *β* = -0.02 [-0.27, 0.23]  16-Choice versus 4-Choice * HRFS: *β* = 0.15 [-0.08, 0.38] | 16-Choice versus 1-Choice * HRFS: *p* = .870  16-Choice versus 4-Choice * HRFS: *p* = .196 |
| Exercise Engagement | 16-Choice versus 1-Choice * HRFS: *β* = -0.07 [-0.34, 0.20]  16-Choice versus 4-Choice * HRFS: *β* = -0.04 [-0.29, 0.20] | 16-Choice versus 1-Choice * HRFS: *p* = .595  16-Choice versus 4-Choice * HRFS: *p* = .728 |
| Attitudes towards Chosen Exercise | 16-Choice versus 1-Choice * HRFS: *β* = 0.00 [-0.27, 0.27]  16-Choice versus 4-Choice * HRFS: *β* = 0.09 [-0.16, 0.34] | 16-Choice versus 1-Choice * HRFS: *p* = .999  16-Choice versus 4-Choice * HRFS: *p* = .479 |
| Perceived Improvement in Mental State | 16-Choice versus 1-Choice * HRFS: *β* = 0.07 [-0.20, 0.35]  16-Choice versus 4-Choice * HRFS: *β* = -0.01 [-0.27, 0.23] | 16-Choice versus 1-Choice * HRFS: *p* = .592  16-Choice versus 4-Choice * HRFS: *p* = .878 |

^a^HRFS refers to Health Regulatory Focus Scale [55]

**Table S4.** Interaction Results (Linear Regressions) with Preference for Choices [42] as the Potential Moderator

| Dependent Variables | *β* [95% CI] | *p* |
| --- | --- | --- |
| Decision Satisfaction | 16-Choice versus 1-Choice * PFC^a^: *β* = -0.13 [-0.32, 0.06]  16-Choice versus 4-Choice * PFC: *β* = -0.11 [-0.31, 0.08] | 16-Choice versus 1-Choice * PFC: *p* = .177  16-Choice versus 4-Choice * PFC: *p* = .236 |
| Chosen Exercise Satisfaction | 16-Choice versus 1-Choice * PFC: *β* = 0.03 [-0.27, 0.23]  16-Choice versus 4-Choice * PFC: *β* = -0.01 [-0.25, 0.22] | 16-Choice versus 1-Choice * PFC: *p* = .777  16-Choice versus 4-Choice * PFC: *p* = .929 |
| Exercise Engagement | 16-Choice versus 1-Choice * PFC: *β* = 0.03 [-0.23, 0.28]  16-Choice versus 4-Choice * PFC: *β* = 0.05 [-0.20, 0.30] | 16-Choice versus 1-Choice * PFC: *p* = .844  16-Choice versus 4-Choice * PFC: *p* = .699 |
| Attitudes towards Chosen Exercise | 16-Choice versus 1-Choice * PFC: *β* = -0.01 [-0.27, 0.24]  16-Choice versus 4-Choice * PFC: *β* = -0.09 [-0.33, 0.16] | 16-Choice versus 1-Choice * PFC: *p* = .909  16-Choice versus 4-Choice * PFC: *p* = .478 |
| Perceived Improvement in Mental State | 16-Choice versus 1-Choice * PFC: *β* = 0.06 [-0.19, 0.32]  16-Choice versus 4-Choice * PFC: *β* = -0.03 [-0.28, 0.22] | 16-Choice versus 1-Choice * PFC: *p* = .624  16-Choice versus 4-Choice * PFC: *p* = .801 |

^a^PFC refers to Preference for Choices [42].

**Table S5.** Interaction Results (Linear Regressions) with Perceived Difficulty of Making Choices [42] as the Potential Moderator

| Dependent Variables | *β* [95% CI] | *p* |
| --- | --- | --- |
| Decision Satisfaction | 16-Choice versus 1-Choice * PDMC^c^: *β* = 0.10 [-0.09, 0.28]  16-Choice versus 4-Choice * PDMC: *β* = 0.04 [-0.16, 0.23] | 16-Choice versus 1-Choice * PDMC: *p* = .307  16-Choice versus 4-Choice * PDMC: *p* = .707 |
| Chosen Exercise Satisfaction | 16-Choice versus 1-Choice * PDMC: *β* = -0.03 [-0.27, 0.20]  16-Choice versus 4-Choice * PDMC: *β* = -0.07 [-0.30, 0.17] | 16-Choice versus 1-Choice * PDMC: *p* = .780  16-Choice versus 4-Choice * PDMC: *p* = .576 |
| Exercise Engagement | 16-Choice versus 1-Choice * PDMC: *β* = -0.02 [-0.28, 0.23]  16-Choice versus 4-Choice * PDMC: *β* = -0.20 [-0.44, 0.05] | 16-Choice versus 1-Choice * PDMC: *p* = .846  16-Choice versus 4-Choice * PDMC: *p* = .120 |
| Attitudes towards Chosen Exercise | 16-Choice versus 1-Choice * PDMC: *β* = 0.06 [-0.20, 0.31]  16-Choice versus 4-Choice * PDMC: *β* = -0.10 [-0.35, 0.15] | 16-Choice versus 1-Choice * PDMC: *p* = .668  16-Choice versus 4-Choice * PDMC: *p* = .432 |
| Perceived Improvement in Mental State | 16-Choice versus 1-Choice * PDMC: *β* = 0.11 [-0.15, 0.36]  16-Choice versus 4-Choice * PDMC: *β* = 0.01 [-0.24, 0.26] | 16-Choice versus 1-Choice * PDMC: *p* = .411  16-Choice versus 4-Choice * PDMC: *p* = .923 |

^c^PDMC refers to Perceived Difficulty of Making Choices [42].

**Supplementary Analyses with Categorical State Emotions as Potential Moderator**

Another way of conducting regression analyses with state emotions is through categorizing participants who experienced more intense unpleasant emotions as one group, and participants who experienced more intense pleasant emotions as another group, so state emotions would be a categorical variable. Results are generally similar to the method reported in the main manuscript. That said, the planned contrasts for attitudes towards chosen exercise had several p-values close to but above .05, whereas p-values at -1SD (through simple slope analyses) fell below .05 for the method reported in the main manuscript. We believe the method reported in the main manuscript is more appropriate, as that method retains more statistical power without excluding participants with equal intensity of pleasant and unpleasant emotions, while quantifying the intensity of pleasant emotions and unpleasant emotions better with a continuum of participants instead of dichotomization. Nonetheless, we report results of the alternative method for transparency purposes in the following.

**Table S6.** Interaction Results (Linear Regressions) with State Emotions (Categorical: Stronger Pleasant or Stronger Unpleasant) as the Potential Moderator

| Dependent Variables | *β* [95% CI] | *p* |
| --- | --- | --- |
| Decision Satisfaction | 16-Choice versus 1-Choice * State Emotions: *β* = 0.35 [-0.04, 0.75]  16-Choice versus 4-Choice * State Emotions: *β* = 0.00 [-0.39, 0.39] | 16-Choice versus 1-Choice * State Emotions: *p* = .080  16-Choice versus 4-Choice * State Emotions: *p* = .993 |
| Chosen Exercise Satisfaction | 16-Choice versus 1-Choice * State Emotions: *β* = 0.85 [0.36, 1.34]  16-Choice versus 4-Choice * State Emotions: *β* = 0.36 [-0.10, 0.82] | 16-Choice versus 1-Choice * State Emotions: *p* < .001  16-Choice versus 4-Choice * State Emotions: *p* = .129 |
| Exercise Engagement | 16-Choice versus 1-Choice * State Emotions: *β* = 0.34 [-0.20, 0.88]  16-Choice versus 4-Choice * State Emotions: *β* = 0.38 [-0.12, 0.87] | 16-Choice versus 1-Choice * State Emotions: *p* = .220  16-Choice versus 4-Choice * State Emotions: *p* = .139 |
| Attitudes towards Chosen Exercise | 16-Choice versus 1-Choice * State Emotions: *β* = 0.70 [0.15, 1.24]  16-Choice versus 4-Choice * State Emotions: *β* = 0.51 [0.01, 1.01] | 16-Choice versus 1-Choice * State Emotions: *p* = .012  16-Choice versus 4-Choice * State Emotions: *p* = .045 |
| Perceived Improvement in Psychological State | 16-Choice versus 1-Choice * State Emotions: *β* = 0.65 [0.10, 1.19]  16-Choice versus 4-Choice * State Emotions: *β* = 0.30 [-0.21, 0.80] | 16-Choice versus 1-Choice * State Emotions: *p* = .021  16-Choice versus 4-Choice * State Emotions: *p* = .250 |

After that, we conducted comparisons between 16-choice condition and 1-choice condition, as well as 16-choice condition and 4-choice condition, for chosen exercise satisfaction, attitudes towards chosen exercise, and perceived improvement in psychological state, for people categorized as experiencing stronger pleasant emotions and people categorized as experiencing stronger unpleasant emotions. Such results are reported in Table S7.

**Table S7.** Contrasts between Conditions among Participants with Stronger Pleasant Emotions and Participants with Stronger Unpleasant Emotions

| Group | Chosen Exercise Satisfaction | Attitudes towards Chosen Exercise | Perceived Improvement in Psychological State |
| --- | --- | --- | --- |
| Stronger Pleasant Emotions  (Pleasant Emotions Average > Unpleasant Emotions Average) | 16-Choice versus 1-Choice: *d* = -0.31, 95% CI [-0.69, 0.06], *p* = .100  16-Choice versus 4-Choice: *d* = -0.18, 95% CI [-0.52, 0.17], *p* = .309 | 16-Choice versus 1-Choice: *d* = -0.36, 95% CI [-0.76, 0.05], *p* = .087  16-Choice versus 4-Choice: *d* = -0.22, 95% CI [-0.59, 0.15], *p* = .233 | 16-Choice versus 1-Choice: *d* = -0.23, 95% CI [-0.69, 0.06], *p* = .269  16-Choice versus 4-Choice: *d* = -0.05, 95% CI [-0.52, 0.17], *p* = .785 |
| Stronger Unpleasant Emotions (Unpleasant Emotions Average > Pleasant Emotions Average) | 16-Choice versus 1-Choice: *d* = 0.55, 95% CI [0.22, 0.89], *p* = .001  16-Choice versus 4-Choice: *d* = 0.19, 95% CI [-0.13, 0.51], *p* = .254 | 16-Choice versus 1-Choice: *d* = 0.34, 95% CI [-0.02, 0.70], *p* = .062  16-Choice versus 4-Choice: *d* = 0.29, 95% CI [-0.05, 0.62], *p* = .096 | 16-Choice versus 1-Choice: *d* = 0.41, 95% CI [0.05, 0.77], *p* = .025  16-Choice versus 4-Choice: *d* = 0.24, 95% CI [-0.10, 0.58], *p* = .160 |

**Exploratory Analyses with Separate State Emotions Items as Potential Moderators**

We report moderation analyses with each measured state emotion in the following for Chosen Exercise Satisfaction, Engagement, Attitudes towards Chosen Exercise, and Perceived Improvement in Psychological State. For Decision Satisfaction, Practice Decision, and Completion as dependent variables, all results are non-significant.

**Table S8.** Interaction Results of Linear Regressions with All State Emotions Measured as Potential Moderators

| Dependent Variables | *β* [95% CI] | *p* |
| --- | --- | --- |
| Chosen Exercise Satisfaction | 16-Choice versus 1-Choice * Tired: *β* = -0.33 [-0.57, -0.10]  16-Choice versus 4-Choice * Tired: *β* = -0.19 [-0.42, 0.03]  16-Choice versus 1-Choice * Excited: *β* = 0.09 [-0.35, 0.02]  16-Choice versus 4-Choice * Excited: *β* = 0.02 [-0.29, 0.08]  16-Choice versus 1-Choice * Distress: *β* = -0.26 [-0.03, 0.34]  16-Choice versus 4-Choice * Distress: *β* = -0.16 [-0.17, 0.20]  16-Choice versus 1-Choice * Tense: *β* = -0.36 [-0.12, 0.26]  16-Choice versus 4-Choice * Tense: *β* = -0.12 [-0.07, 0.29]  16-Choice versus 1-Choice * Happy: *β* = 0.23 [-0.01, 0.47]  16-Choice versus 4-Choice * Happy: *β* = 0.16 [-0.06, 0.38]  16-Choice versus 1-Choice * Sad: *β* = -0.27 [-0.50, -0.05]  16-Choice versus 4-Choice * Sad: *β* = -0.23 [-0.44, -0.02]  16-Choice versus 1-Choice * Calm: *β* = 0.21 [-0.32, 0.05]  16-Choice versus 4-Choice * Calm: *β* = 0.05 [-0.31, 0.07]  16-Choice versus 1-Choice * Relaxed: *β* = 0.30 [-0.09, 0.27]  16-Choice versus 4-Choice * Relaxed: *β* = 0.19 [-0.17, 0.21] | 16-Choice versus 1-Choice * Tired: *p* = .005 (above adjusted alpha of 0.00125)  16-Choice versus 4-Choice * Tired: *p* = .097  16-Choice versus 1-Choice * Excited: *p* = .433  16-Choice versus 4-Choice * Excited: *p* = .833  16-Choice versus 1-Choice * Distress: *p* = .031 (above adjusted alpha of 0.00375)  16-Choice versus 4-Choice * Distress: *p* = .170  16-Choice versus 1-Choice * Tense: *p* = .002 (above adjusted alpha of 0.000625)  16-Choice versus 4-Choice * Tense: *p* = .274  16-Choice versus 1-Choice * Happy: *p* = .061  16-Choice versus 4-Choice * Happy: *p* = .153  16-Choice versus 1-Choice * Sad: *p* = .015 (above adjusted alpha of 0.0025)  16-Choice versus 4-Choice * Sad: *p* = .032 (above adjusted alpha of 0.004375)  16-Choice versus 1-Choice * Calm: *p* = .085  16-Choice versus 4-Choice * Calm: *p* = .659  16-Choice versus 1-Choice * Relaxed: *p* = .015 (above adjusted alpha of 0.001875)  16-Choice versus 4-Choice * Relaxed: *p* = .097 |
| Exercise Engagement | 16-Choice versus 1-Choice * Tired: *β* = -0.07 [-0.32, 0.18]  16-Choice versus 4-Choice * Tired: *β* = -0.18 [-0.42, 0.06]  16-Choice versus 1-Choice * Excited: *β* = 0.04 [-0.20, 0.29]  16-Choice versus 4-Choice * Excited: *β* = 0.10 [-0.14, 0.34]  16-Choice versus 1-Choice * Distress: *β* = -0.04 [-0.30, 0.22]  16-Choice versus 4-Choice * Distress: *β* = 0.09 [-0.15, 0.34]  16-Choice versus 1-Choice * Tense: *β* = -0.12 [-0.36, 0.13]  16-Choice versus 4-Choice * Tense: *β* = 0.02 [-0.22, 0.25]  16-Choice versus 1-Choice * Happy: *β* = 0.20 [-0.07, 0.47]  16-Choice versus 4-Choice * Happy: *β* = 0.18 [-0.06, 0.42]  16-Choice versus 1-Choice * Sad: *β* = -0.14 [-0.39, 0.10]  16-Choice versus 4-Choice * Sad: *β* = -0.09 [-0.31, 0.14]  16-Choice versus 1-Choice * Calm: *β* = -0.01 [-0.27, 0.25]  16-Choice versus 4-Choice * Calm: *β* = 0.02 [-0.22, 0.26]  16-Choice versus 1-Choice * Relaxed: *β* = 0.27 [0.01, 0.54]  16-Choice versus 4-Choice * Relaxed: *β* = 0.28 [0.04, 0.53] | 16-Choice versus 1-Choice * Tired: *p* = .579  16-Choice versus 4-Choice * Tired: *p* = .134  16-Choice versus 1-Choice * Excited: *p* = .730  16-Choice versus 4-Choice * Excited: *p* = .426  16-Choice versus 1-Choice * Distress: *p* = .761  16-Choice versus 4-Choice * Distress: *p* = .464  16-Choice versus 1-Choice * Tense: *p* = .359  16-Choice versus 4-Choice * Tense: *p* = .885  16-Choice versus 1-Choice * Happy: *p* = .142  16-Choice versus 4-Choice * Happy: *p* = .138  16-Choice versus 1-Choice * Sad: *p* = .241  16-Choice versus 4-Choice * Sad: *p* = .451  16-Choice versus 1-Choice * Calm: *p* = .939  16-Choice versus 4-Choice * Calm: *p* = .873  16-Choice versus 1-Choice * Relaxed: *p* = .044 (above adjusted alpha of 0.005)  16-Choice versus 4-Choice * Relaxed: *p* = .025 (above adjusted alpha of 0.003125) |
| Attitudes towards Chosen Exercise | 16-Choice versus 1-Choice * Tired: *β* = -0.32 [-0.57, -0.07]  16-Choice versus 4-Choice * Tired: *β* = -0.32 [-0.55, -0.08]  16-Choice versus 1-Choice * Excited: *β* = 0.08 [-0.17, 0.33]  16-Choice versus 4-Choice * Excited: *β* = 0.05 [-0.19, 0.29]  16-Choice versus 1-Choice * Distress: *β* = -0.14 [-0.40, 0.11]  16-Choice versus 4-Choice * Distress: *β* = -0.12 [-0.37, 0.13]  16-Choice versus 1-Choice * Tense: *β* = -0.29 [-0.54, -0.04]  16-Choice versus 4-Choice * Tense: *β* = -0.12 [-0.36, 0.11]  16-Choice versus 1-Choice * Happy: *β* = 0.25 [-0.02, 0.51]  16-Choice versus 4-Choice * Happy: *β* = 0.23 [-0.01, 0.47]  16-Choice versus 1-Choice * Sad: *β* = -0.24 [-0.48, -0.00]  16-Choice versus 4-Choice * Sad: *β* = -0.25 [-0.47, -0.03]  16-Choice versus 1-Choice * Calm: *β* = 0.05 [-0.22, 0.31]  16-Choice versus 4-Choice * Calm: *β* = 0.03 [-0.21, 0.27]  16-Choice versus 1-Choice * Relaxed: *β* = 0.34 [0.08, 0.60]  16-Choice versus 4-Choice * Relaxed: *β* = 0.33 [0.08, 0.58] | 16-Choice versus 1-Choice * Tired: *p* = .011 (above adjusted alpha of 0.0078125)  16-Choice versus 4-Choice * Tired: *p* = .009 (above adjusted alpha of 0.0046875)  16-Choice versus 1-Choice * Excited: *p* = .524  16-Choice versus 4-Choice * Excited: *p* = .660  16-Choice versus 1-Choice * Distress: *p* = .278  16-Choice versus 4-Choice * Distress: *p* = .338  16-Choice versus 1-Choice * Tense: *p* = .021 (above adjusted alpha of 0.009375)  16-Choice versus 4-Choice * Tense: *p* = .309  16-Choice versus 1-Choice * Happy: *p* = .073  16-Choice versus 4-Choice * Happy: *p* = .058  16-Choice versus 1-Choice * Sad: *p* = .048  16-Choice versus 4-Choice * Sad: *p* = .025 (above adjusted alpha of 0.0109375)  16-Choice versus 1-Choice * Calm: *p* = .719  16-Choice versus 4-Choice * Calm: *p* = .796  16-Choice versus 1-Choice * Relaxed: *p* = .011 (above adjusted alpha of 0.00625)  16-Choice versus 4-Choice * Relaxed: *p* = .009 (above adjusted alpha of 0.003125) |
| Perceived Improvement in Psychological State | 16-Choice versus 1-Choice * Tired: *β* = -0.22 [-0.47, 0.03]  16-Choice versus 4-Choice * Tired: *β* = -0.24 [-0.48, 0.01]  16-Choice versus 1-Choice * Excited: *β* = -0.06 [-0.30, 0.19]  16-Choice versus 4-Choice * Excited: *β* = -0.13 [-0.37, 0.10]  16-Choice versus 1-Choice * Distress: *β* = -0.22 [-0.47, 0.04]  16-Choice versus 4-Choice * Distress: *β* = -0.15 [-0.40, 0.10]  16-Choice versus 1-Choice * Tense: *β* = -0.26 [-0.51, -0.01]  16-Choice versus 4-Choice * Tense: *β* = -0.12 [-0.36, 0.12]  16-Choice versus 1-Choice * Happy: *β* = 0.22 [-0.05, 0.49]  16-Choice versus 4-Choice * Happy: *β* = 0.01 [-0.23, 0.25]  16-Choice versus 1-Choice * Sad: *β* = -0.42 [-0.66, -0.18]  16-Choice versus 4-Choice * Sad: *β* = -0.22 [-0.44, -0.00]  16-Choice versus 1-Choice * Calm: *β* = 0.07 [-0.20, 0.34]  16-Choice versus 4-Choice * Calm: *β* = 0.02 [-0.23, 0.26]  16-Choice versus 1-Choice * Relaxed: *β* = 0.18 [-0.08, 0.45]  16-Choice versus 4-Choice * Relaxed: *β* = 0.20 [-0.04, 0.45] | 16-Choice versus 1-Choice * Tired: *p* = .080  16-Choice versus 4-Choice * Tired: *p* = .055  16-Choice versus 1-Choice * Excited: *p* = .658  16-Choice versus 4-Choice * Excited: *p* = .266  16-Choice versus 1-Choice * Distress: *p* = .096  16-Choice versus 4-Choice * Distress: *p* = .228  16-Choice versus 1-Choice * Tense: *p* = .040 (above adjusted alpha of 0.0125)  16-Choice versus 4-Choice * Tense: *p* = .324  16-Choice versus 1-Choice * Happy: *p* = .110  16-Choice versus 4-Choice * Happy: *p* = .946  16-Choice versus 1-Choice * Sad: *p* < .001 (below adjusted alpha of 0.0015625)  16-Choice versus 4-Choice * Sad: *p* = .045 (above adjusted alpha of 0.0140625)  16-Choice versus 1-Choice * Calm: *p* = .602  16-Choice versus 4-Choice * Calm: *p* = .887  16-Choice versus 1-Choice * Relaxed: *p* = .170  16-Choice versus 4-Choice * Relaxed: *p* = .103 |

**Analyses without Excluding Any Participants who Did Not Pass Both Validation Checks**

**Table S9.** Overall Comparison Between Different Numbers of Choice(s)

|  | 16-Choice versus 1-Choice | 4-Choice versus 1-Choice | 16-Choice versus 4-Choice |
| --- | --- | --- | --- |
| Practice Decision | *OR* = 4.11, 95% CI [2.74, 6.16], *p* < .001 | *OR* = 3.31, 95% CI [2.21, 4.95], *p* < .001 | *OR* = 1.24, 95% CI [0.86, 1.80], *p* = .253 |
| Practice Completion | *OR* = 0.69, 95% CI [0.38, 1.26], *p* = .232 | *OR* = 1.01, 95% CI [0.54, 1.89], *p* = .984 | *OR* = 0.69, 95% CI [0.39, 1.22], *p* = .202 |
| Decision Satisfaction | *t*(440.31) = 3.90, *d* = 0.37, 95% CI [0.18, 0.56], *p* < .001 | *t*(442.97) = 2.62, *d* = 0.25, 95% CI [0.06, 0.44], *p* = .025 | *t*(439.83) = 1.37, *d* = 0.13, 95% CI [-0.06, 0.32], *p* = .358 |
| Chosen Exercise Satisfaction | *t*(288.85) = 1.84, *d* = 0.22, 95% CI [-0.02, 0.45], *p* = .160 | *t*(283.14) = 1.01, *d* = 0.12, 95% CI [-0.11, 0.35], *p* = .569 | *t*(312.95) = 0.85, *d* = 0.10, 95% CI [-0.13, 0.32], *p* = .669 |
| Exercise Engagement | *t*(243.09) = -0.85, *d* = -0.11, 95% CI [-0.36, 0.14], *p* = .673 | *t*(246.87) = -1.13, *d* = -0.14, 95% CI [-0.39, 0.11], *p* = .495 | *t*(274.41) = 0.35, *d* = 0.04, 95% CI [-0.19, 0.28], *p* = .669 |
| Attitudes towards Chosen Exercise | *t*(240.18) = 0.48, *d* = 0.06, 95% CI [-0.19, 0.32], *p* = .879 | *t*(237.01) = -0.02, *d* = -0.00, 95% CI [-0.26, 0.25], *p* = 1.00 | *t*(276.86) = 0.53, *d* = 0.00, 95% CI [-0.23, 0.24], *p* = .856 |
| Perceived Improvement in Psychological State | *t*(239.17) = 1.01, *d* = 0.13, 95% CI [-0.12, 0.38], *p* = .574 | *t*(233.49) = 0.05, *d* = 0.00, 95% CI [-0.25, 0.26], *p* = .998 | *t*(276.79) = 1.01, *d* = 0.12, 95% CI [-0.11, 0.36], *p* = .570 |

**Table S10.** Interaction Results (Linear Regressions) (including participants who did not pass any validation check)

| Dependent Variables | *β* [95% CI] | *p* |
| --- | --- | --- |
| Decision Satisfaction | 16-Choice versus 1-Choice * PHQ: *β* = -0.04 [-0.24, 0.16]  16-Choice versus 4-Choice * PHQ: *β* = 0.02 [-0.18, 0.21]  16-Choice versus 1-Choice * GAD: *β* = -0.16 [-0.35, 0.02]  16-Choice versus 4-Choice * GAD: *β* = -0.10 [-0.29, 0.08]  16-Choice versus 1-Choice * State Emotions: *β* = 0.15 [-0.03, 0.34]  16-Choice versus 4-Choice * State Emotions: *β* = 0.02 [-0.17, 0.20]  16-Choice versus 1-Choice * HRFS: *β* = 0.07 [-0.12, 0.26]  16-Choice versus 4-Choice * HRFS: *β* = 0.11 [-0.07, 0.29]  16-Choice versus 1-Choice * PFC: *β* = -0.13 [-0.32, 0.05]  16-Choice versus 4-Choice * PFC: *β* = -0.12 [-0.31, 0.07]  16-Choice versus 1-Choice * PDMC: *β* = 0.09 [-0.09, 0.27]  16-Choice versus 4-Choice * PMDC: *β* = 0.02 [-0.17, 0.21] | 16-Choice versus 1-Choice * PHQ: *p* = .665  16-Choice versus 4-Choice * PHQ: *p* = .842  16-Choice versus 1-Choice * GAD: *p* = .096  16-Choice versus 4-Choice * GAD:  *p* = .280  16-Choice versus 1-Choice * State Emotions: *p* = .103  16-Choice versus 4-Choice * State Emotions: *p* = .857  16-Choice versus 1-Choice * HRFS: *p* = .468  16-Choice versus 4-Choice * HRFS: *p* = .221  16-Choice versus 1-Choice * PFC: *p* = .159  16-Choice versus 4-Choice * PFC: *p* = .205  16-Choice versus 1-Choice * PDMC: *p* = .331  16-Choice versus 4-Choice * PDMC: *p* = .819 |
| Chosen Exercise Satisfaction | 16-Choice versus 1-Choice * PHQ: *β* = -0.15 [-0.39, 0.10]  16-Choice versus 4-Choice * PHQ: *β* = -0.17 [-0.40, 0.06]  16-Choice versus 1-Choice * GAD: *β* = -0.27 [-0.50, -0.05]  16-Choice versus 4-Choice * GAD: *β* = -0.24 [-0.45, -0.02]  16-Choice versus 1-Choice * State Emotions: *β* = 0.37 [0.15, 0.60]  16-Choice versus 4-Choice * State Emotions: *β* = 0.23 [0.01, 0.44]  16-Choice versus 1-Choice * HRFS: *β* = -0.06 [-0.30, 0.19]  16-Choice versus 4-Choice * HRFS: *β* = 0.13 [-0.09, 0.35]  16-Choice versus 1-Choice * PFC: *β* = 0.02 [-0.22, 0.26]  16-Choice versus 4-Choice * PFC: *β* = -0.02 [-0.25, 0.21]  16-Choice versus 1-Choice * PDMC: *β* = -0.03 [-0.26, 0.20]  16-Choice versus 4-Choice * PMDC: *β* = -0.08 [-0.31, 0.15] | 16-Choice versus 1-Choice * PHQ: *p* = .234  16-Choice versus 4-Choice * PHQ: *p* = .155  16-Choice versus 1-Choice * GAD: *p* = .016  16-Choice versus 4-Choice * GAD:  *p* = .030  16-Choice versus 1-Choice * State Emotions: *p* = .001  16-Choice versus 4-Choice * State Emotions: *p* = .038  16-Choice versus 1-Choice * HRFS: *p* = .640  16-Choice versus 4-Choice * HRFS: *p* = .250  16-Choice versus 1-Choice * PFC: *p* = .863  16-Choice versus 4-Choice * PFC: *p* = .879  16-Choice versus 1-Choice * PDMC: *p* = .797  16-Choice versus 4-Choice * PDMC: *p* = .511 |
| Exercise Engagement | 16-Choice versus 1-Choice * PHQ: *β* = -0.02 [-0.28, 0.25]  16-Choice versus 4-Choice * PHQ: *β* = -0.04 [-0.30, 0.21]  16-Choice versus 1-Choice * GAD: *β* = -0.07 [-0.32, 0.18]  16-Choice versus 4-Choice * GAD: *β* = -0.03 [-0.27, 0.20]  16-Choice versus 1-Choice * State Emotions: *β* = 0.13 [-0.12, 0.39]  16-Choice versus 4-Choice * State Emotions: *β* = 0.13 [-0.11, 0.37]  16-Choice versus 1-Choice * HRFS: *β* = -0.08 [-0.26, 0.13]  16-Choice versus 4-Choice * HRFS: *β* = -0.03 [-0.18, 0.20]  16-Choice versus 1-Choice * PFC: *β* = 0.02 [-0.23, 0.27]  16-Choice versus 4-Choice * PFC: *β* = 0.05 [-0.19, 0.29]  16-Choice versus 1-Choice * PDMC: *β* = 0.01 [-0.24, 0.25]  16-Choice versus 4-Choice * PMDC: *β* = -0.18 [-0.42, 0.07] | 16-Choice versus 1-Choice * PHQ: *p* = .907  16-Choice versus 4-Choice * PHQ: *p* = .725  16-Choice versus 1-Choice * GAD: *p* = .589  16-Choice versus 4-Choice * GAD:  *p* = .776  16-Choice versus 1-Choice * State Emotions: *p* = .305  16-Choice versus 4-Choice * State Emotions: *p* = .278  16-Choice versus 1-Choice * HRFS: *p* = .555  16-Choice versus 4-Choice * HRFS: *p* = .822  16-Choice versus 1-Choice * PFC: *p* = .889  16-Choice versus 4-Choice * PFC: *p* = .690  16-Choice versus 1-Choice * PDMC: *p* = .966  16-Choice versus 4-Choice * PDMC: *p* = .155 |
| Attitudes towards Chosen Exercise | 16-Choice versus 1-Choice * PHQ: *β* = -0.10 [-0.37, 0.16]  16-Choice versus 4-Choice * PHQ: *β* = -0.18 [-0.43, 0.07]  16-Choice versus 1-Choice * GAD: *β* = -0.29 [-0.54, -0.04]  16-Choice versus 4-Choice * GAD: *β* = -0.26 [-0.50, -0.02]  16-Choice versus 1-Choice * State Emotions: *β* = 0.32 [0.07, 0.57]  16-Choice versus 4-Choice * State Emotions: *β* = 0.27 [0.03, 0.50]  16-Choice versus 1-Choice * HRFS: *β* = -0.02 [-0.28, 0.24]  16-Choice versus 4-Choice * HRFS: *β* = 0.09 [-0.15, 0.33]  16-Choice versus 1-Choice * PFC: *β* = -0.03 [-0.28, 0.22]  16-Choice versus 4-Choice * PFC: *β* = -0.11 [-0.35, 0.14]  16-Choice versus 1-Choice * PDMC: *β* = 0.08 [-0.17, 0.33]  16-Choice versus 4-Choice * PMDC: *β* = -0.09 [-0.34, 0.16] | 16-Choice versus 1-Choice * PHQ: *p* = .445  16-Choice versus 4-Choice * PHQ: *p* = .166*  16-Choice versus 1-Choice * GAD: *p* = .023  16-Choice versus 4-Choice * GAD:  *p* = .032  16-Choice versus 1-Choice * State Emotions: *p* = .013  16-Choice versus 4-Choice * State Emotions: *p* = .026  16-Choice versus 1-Choice * HRFS: *p* = .883  16-Choice versus 4-Choice * HRFS: *p* = .444  16-Choice versus 1-Choice * PFC: *p* = .821  16-Choice versus 4-Choice * PFC: *p* = .393  16-Choice versus 1-Choice * PDMC: *p* = .522  16-Choice versus 4-Choice * PDMC: *p* = .467 |
| Perceived Improvement in Psychological State | 16-Choice versus 1-Choice * PHQ: *β* = -0.20 [-0.47, 0.07]  16-Choice versus 4-Choice * PHQ: *β* = -0.19 [-0.45, 0.06]  16-Choice versus 1-Choice * GAD: *β* = -0.33 [-0.58, -0.08]  16-Choice versus 4-Choice * GAD: *β* = -0.27 [-0.51, -0.04]  16-Choice versus 1-Choice * State Emotions: *β* = 0.30 [0.05, 0.55]  16-Choice versus 4-Choice * State Emotions: *β* = 0.17 [-0.06, 0.41]  16-Choice versus 1-Choice * HRFS: *β* = 0.05 [-0.22, 0.32]  16-Choice versus 4-Choice * HRFS: *β* = -0.03 [-0.27, 0.22]  16-Choice versus 1-Choice * PFC: *β* = 0.06 [-0.19, 0.31]  16-Choice versus 4-Choice * PFC: *β* = -0.04 [-0.28, 0.21]  16-Choice versus 1-Choice * PDMC: *β* = 0.12 [-0.13, 0.37]  16-Choice versus 4-Choice * PMDC: *β* = 0.01 [-0.23, 0.26] | 16-Choice versus 1-Choice * PHQ: *p* = .144  16-Choice versus 4-Choice * PHQ: *p* = .128  16-Choice versus 1-Choice * GAD: *p* = .010  16-Choice versus 4-Choice * GAD:  *p* = .025  16-Choice versus 1-Choice * State Emotions: *p* = .020  16-Choice versus 4-Choice * State Emotions: *p* = .145  16-Choice versus 1-Choice * HRFS: *p* = .722  16-Choice versus 4-Choice * HRFS: *p* = .836  16-Choice versus 1-Choice * PFC: *p* = .658  16-Choice versus 4-Choice * PFC: *p* = .763  16-Choice versus 1-Choice * PDMC: *p* = .339  16-Choice versus 4-Choice * PDMC: *p* = .921 |

*Given that we found no support for moderation (p = .166) while including participants who did not pass both validation checks, we are uncertain regarding the possible moderation effect we found tentative evidence for in the main results that excluded participants who failed validation checks.

**Table S11.** Simple Slope Analyses with Chosen Exercise Satisfaction, Attitudes towards Chosen Exercise, and Perceived Improvement in Psychological State, with GAD-9 as Potential Moderator (not excluding participants who did not pass any of the validation checks)

| GAD-9 Level | Chosen Exercise Satisfaction | Attitudes towards Chosen Exercise | Perceived Improvement in Psychological State |
| --- | --- | --- | --- |
| -1SD (Lower Anxiety) | 1-Choice versus 16-Choice: *β* = 0.08 [-0.25, 0.41], *p* = .634  4-Choice versus 16-Choice: *β* = 0.14 [-0.17, 0.46], *p* = .368 | 1-Choice versus 16-Choice: *β* = 0.24 [-0.12, 0.60], *p* = .187  4-Choice versus 16-Choice: *β* = 0.20 [-0.14, 0.53], *p* = .248 | 1-Choice versus 16-Choice: *β* = 0.21 [-0.15, 0.57], *p* = .248  4-Choice versus 16-Choice: *β* = 0.15 [-0.18, 0.49], *p* = .374 |
| Mean | 1-Choice versus 16-Choice: *β* = -0.21 [-0.43, 0.02], *p* = .077  4-Choice versus 16-Choice: *β* = -0.10 [-0.32, 0.11], *p* = .349 | 1-Choice versus 16-Choice: *β* = -0.06 [-0.30, 0.19], *p* = .658  4-Choice versus 16-Choice: *β* = -0.07 [-0.30, 0.17], *p* = .574 | 1-Choice versus 16-Choice: *β* = -0.12 [-0.37, 0.12], *p* = .322  4-Choice versus 16-Choice: *β* = -0.12 [-0.36, 0.11], *p* = .296 |
| +1SD (Higher Anxiety) | 1-Choice versus 16-Choice: *β* = -0.49 [-0.81, -0.17], *p* = .003  4-Choice versus 16-Choice: *β* = -0.35 [-0.67, -0.04], *p* = .028 | 1-Choice versus 16-Choice: *β* = -0.35 [-0.70, -0.00], *p* = .050  4-Choice versus 16-Choice: *β* = -0.33 [-0.67, 0.01], *p* = .054 | 1-Choice versus 16-Choice: *β* = -0.46 [-0.81, -0.11], *p* = .011 4-Choice versus 16-Choice: *β* = -0.40 [-0.73, -0.07], *p* = .019 |

**Table S12.** Simple Slope Analyses with Chosen Exercise Satisfaction, Attitudes towards Chosen Exercise, and Perceived Improvement in Psychological State, with State Emotions as Potential Moderator (not excluding participants who did not pass any of the validation checks)

| GAD-9 Level | Chosen Exercise Satisfaction | Attitudes towards Chosen Exercise | Perceived Improvement in Psychological State |
| --- | --- | --- | --- |
| -1SD (Stronger Unpleasant Emotions) | 1-Choice versus 16-Choice: *β* = -0.58 [-0.90, -0.27], *p* < .001  4-Choice versus 16-Choice: *β* = -0.34 [-0.65, -0.02], *p* = .035 | 1-Choice versus 16-Choice: *β* = -0.37 [-0.72, -0.02], *p* = .037 4-Choice versus 16-Choice: *β* = -0.34 [-0.68, -0.00], *p* = .048 | 1-Choice versus 16-Choice: *β* = -0.42 [-0.77, -0.07], *p* = .019  4-Choice versus 16-Choice: *β* = -0.30 [-0.64, 0.03], *p* = .078 |
| Mean | 1-Choice versus 16-Choice: *β* = -0.20 [-0.43, 0.03], *p* = .086  4-Choice versus 16-Choice: *β* = -0.10 [-0.32, 0.11], *p* = .351 | 1-Choice versus 16-Choice: *β* = -0.05 [-0.30, 0.20], *p* = .689  4-Choice versus 16-Choice: *β* = -0.07 [-0.30, 0.16], *p* = .552 | 1-Choice versus 16-Choice: *β* = -0.11 [-0.36, 0.13], *p* = .370  4-Choice versus 16-Choice: *β* = -0.13 [-0.36, 0.11], *p* = .289 |
| +1SD (Stronger Pleasant Emotions) | 1-Choice versus 16-Choice: *β* = 0.19 [-0.14, 0.52], *p* = .266  4-Choice versus 16-Choice: *β* = 0.13 [-0.18, 0.44], *p* = .404 | 1-Choice versus 16-Choice: *β* = 0.27 [-0.09, 0.63], *p* = .137  4-Choice versus 16-Choice: *β* = 0.20 [-0.13, 0.53], *p* = .236 | 1-Choice versus 16-Choice: *β* = 0.19 [-0.17, 0.56], *p* = .295 4-Choice versus 16-Choice: *β* = 0.05 [-0.28, 0.38], *p* = .764 |

**List of Additional Measures in the Data Collection**

To be cost-efficient, the same data collection can serve multiple purposes, including primary confirmatory analyses (with confirmatory moderators reported in the main manuscript) and secondary analyses such as exploratory moderation tests and scale shortening. In line with efforts of sharing large datasets publicly [1-3], aiming to facilitate secondary analyses by different researchers, we reported additional measures included in our data collection in the following and the dataset with such variables is publicly available on OSF (https://osf.io/puwxb/). Particularly, we believe there is potential for exploratory moderation analyses with machine learning methods. In the personalized mental health literature, some researchers have tested and compared numerous potential moderators through machine learning methods [4,5].

1. Additional regulatory focus measures for scale validation and shortening purposes, including Higgins et al [6] Regulatory Focus Questionnaire (RFQ), Lockwood et al [7] General Regulatory Focus Measure (GRFM), Single-Item Mental Health Regulatory Focus, 0-100 Scale Perceived Importance of Mental Health Promotion and Prevention Goals (non-pre-registered)
2. Cacioppo et al [8] Need for Cognition (NFC) scale
3. Mental Health Literacy (self-adapted) [9]
4. Subjective Knowledge of Mental Health (adapted from Hadar & Sood [10])
5. Self-Awareness of Mental Health Needs (self-developed)
6. Analysis-Holism scale [11]
7. Behavior Identification Form [12]
8. Maximization Scale Short Form [13] (only including High Standards and Alternative Search Subscales) and Less Ambitious Satisficing Subscale [14] (non-pre-registered)

While our pre-registered hypotheses focus on practice decision, completion, engagement, satisfaction and we pre-registered and included a few exploratory variables including attitudes towards chosen exercise and perceived psychological state improvement that are reported in the main manuscript, we included several additional measures:

1. Behavioral intention of practicing the exercise in the future (without specifying the timeframe)
2. Behavioral intention of engaging in Jockey Club Tour Heart, a digital mental health platform with self-care exercises.
3. Overload feelings (i.e. confusion, feeling overwhelmed, feeling difficulty in making the decision) [15-17] (non-pre-registered)

**Deviations from Preregistration**

1. As mentioned in the main manuscript, while we pre-registered the Holm alpha adjustment method [18] for confirmatory hypotheses, we did not pre-register alpha adjustment method for exploratory tests. Given a substantial number of exploratory tests conducted and to manage risks of false positives, we decided to adopt Benjamini-Hochberg alpha adjustment method.
2. In the pre-registration, we did not mention the results of validation check as an inclusion-exclusion criteria. We decided to implement validation check results as an inclusion-exclusion criteria to enhance analyzed data quality. We only included participants who passed at least 1 validation check in the main manuscript, but reported participants who did not pass this criteria in the supplementary. The results before and after exclusion are highly similar. The one key difference is that, after exclusion of participants who did not pass any of the validation check, we found tentative evidence (with p-values below .05 but above adjusted alpha) for moderation of depressive symptoms with attitudes towards chosen exercise as dependent variable. However, before the exclusion, we found no evidence for such moderation. More importantly, our key findings of 1) overall differences between conditions in practice decision likelihood and decision satisfaction, 2) tentative evidence for moderation of anxiety and state emotions with chosen exercise satisfaction, attitudes towards chosen exercise, and perceived improvement in psychological state, remain consistent regardless of the inclusion-exclusion criteria.

Additional Note: We forgot to clarify in the pre-registration regarding the specifics of the two-week follow-up survey, with items (dependent variables) highly similar to the first survey as well as items regarding payment information. Since the vast majority (over 85%) of participants did not practice after the first survey, an insufficient number of responses (from <80 participants) on variables such as satisfaction, engagement and attitudes can be meaningfully and reliably analyzed. Such responses are included in the shared datasets (including the cleaned and combined dataset), and other researchers may analyze such data, while understanding the underpowered nature for such variables.

**Details regarding Different Choice Sets and Exercises**

We provide the screenshots of different choice set conditions on Qualtrics in the following. We present both the computer versions and the phone versions. The order of exercises presented were randomized. For the 16-Choice Condition and the 4-Choice Condition, the numbers of audio/video activities and written activities are even. For all conditions, participants were given the options of “choose later” and “do not want to practice”. English versions are provided after the screenshots.

**16-Choice Condition**

**
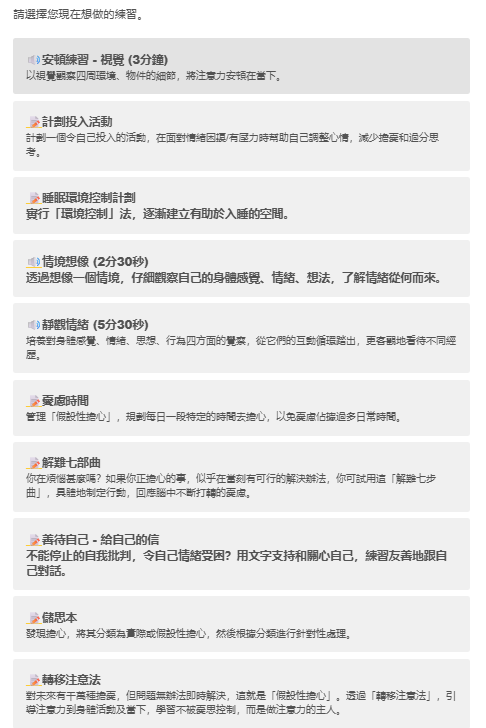

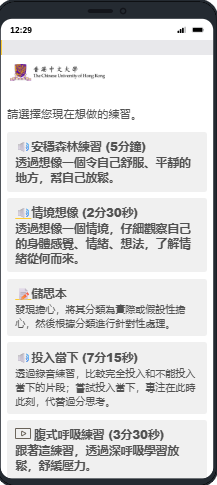
**

**
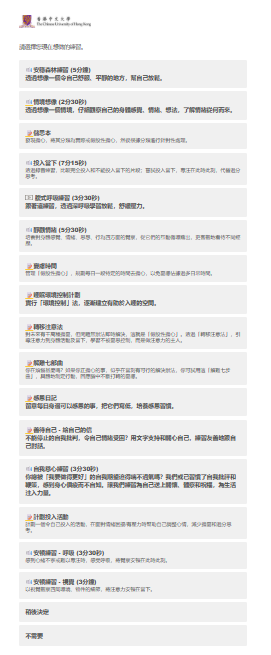
**

**4-Choice Condition**

**
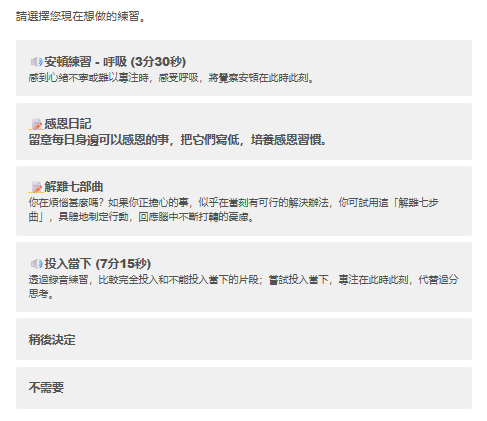

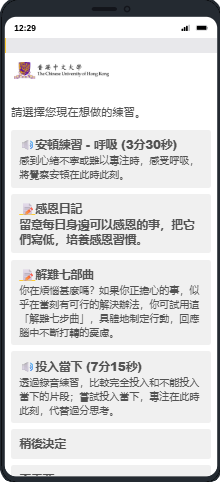
**

Participants were presented with 4 self-care exercise choices in the 4-choice condition. We randomized participants to 1 out of 15 combinations of 4-choice conditions, in which 2 activities involve written exercises and 2 activities involve audio/video exercises for each combination, were randomly selected using a random number generator.

**1-Choice Condition**

**
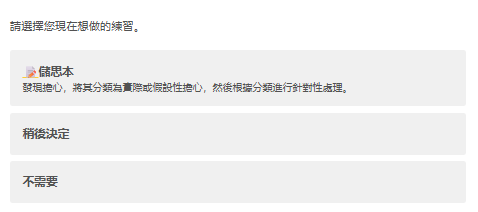

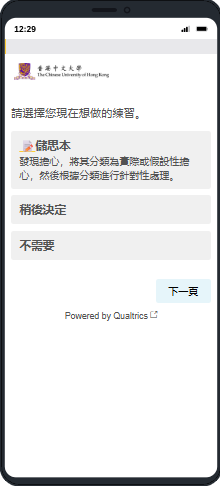
**

Participants were randomized to 1 out of 16 self-care activities used in the 16-choice condition.

**List and Descriptions of Self-Care Exercises (exercises were presented in Chinese in the experiment)**

The following titles and descriptions are translations adapted from the Jockey Club Tour Heart website or based on Poe.

- **Grounding Exercise (Visual) (3 min)**

Observe and be mindful of the surroundings, settling our attention in the present moment through observing with our vision.

- **Generate an Absorbing Activity**

Plan an absorbing activity to regulate your emotions and reduce worry and rumination when under stress or experiencing psychological distress.

- **Building a Sleep-Conducive Environment**

To build a more sleep-conducive environment through stimulus control techniques.

- **Visualization (2m30s)**

Through visualizing a situation, we observe our bodily sensations, emotions, and thoughts to understand the origins of our mental experiences.

- **Mindful Awareness of Emotions (5m30s)**

Develop awareness of thoughts, feelings, and behaviors in order not to be carried away by our vicious cycle of be able to observe our experiences objectively.

- **Worry Time**

Schedule a specific time each day to focus on worries about situations that have not happened yet, so as to prevent worries from occupying too much of daily life.

- **7 Steps of Problem Solving**

Create a specific plan of action and address the worries on issues that may have plausible solutions to deal with.

- **A Kind Letter to Myself**

Write a letter to support and care for yourself and practice speaking kindly to yourself so you can stop self-criticizing.

- **Worry Diary**

Discover your worries, classify them into practical or hypothetical worries, and then cope with them according to the classification.

- **Attention Shifting**

Rather than being bogged down by worries on issues that may not happen, guide our attention to various activities and the present moment, learn not to be controlled by worries but to be the master of attention.

- **Diaphragmatic Breathing (3m30s)**

LLearn to relax and relieve stress through deep breathing.

- **Calm Forest Practice (5 min)**

Imagine a place that is comfortable and calm to help us relax.

- **Gratitude Journal**

Learn to pay attention to the things around you that you are thankful for each day, write them down, and cultivate the habit of gratitude.

- **Mindful Breathing (3m30s)**

Learn to observe the breaths and let awareness settle in the present moment.

- **Self-Kindness Meditation (3m30s)**

Practice giving ourselves care, understanding, and blessings, infusing our lifewith strength and energy, rather than being bogged down by our expectations and criticisms.

- **Connecting with the Present (7m15s)**

Learn to compare between fully vs. not fully absorbed moments and connect with the present moment to replace ruminations.

**Study Flow Chart**

We created this flow chart, through adapting APA JARS Flow Chart: <https://apastyle.apa.org/jars/jars-quant-participant-flowchart.pdf>


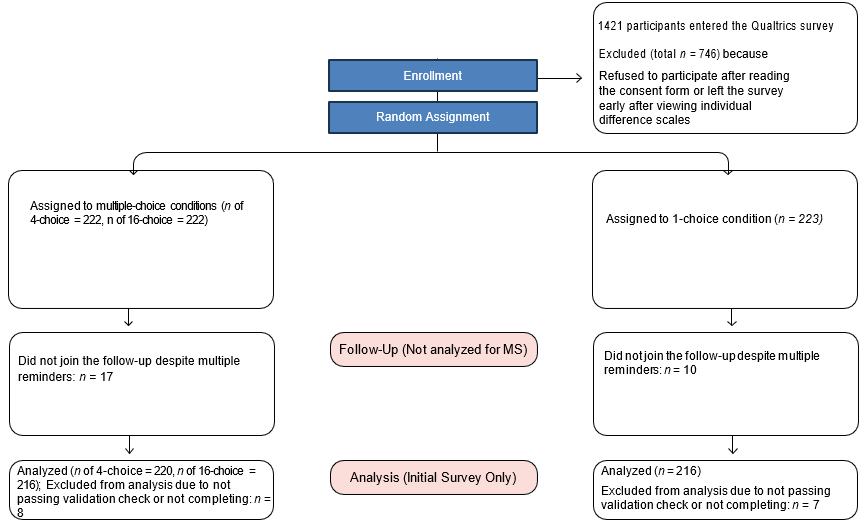


**Supplementary References**

1. Yamada Y, Ćepulić DB, Coll-Martín T, Debove S, Gautreau G, Han H, Rasmussen J, Tran TP, Travaglino GA, Lieberoth A. COVIDiSTRESS Global Survey dataset on psychological and behavioural consequences of the COVID-19 outbreak. Scientific data. 2021 Jan 4;8(1):3.

2. Azevedo F, Pavlović T, Rêgo GG, Ay FC, Gjoneska B, Etienne TW, Ross RM, Schönegger P, Riaño-Moreno JC, Cichocka A, Capraro V. Social and moral psychology of COVID-19 across 69 countries. Scientific data. 2023 May 11;10(1):272.

3. Blackburn AM, Vestergren S. COVIDiSTRESS diverse dataset on psychological and behavioural outcomes one year into the COVID-19 pandemic. Scientific data. 2022 Jun 21;9(1):331.

4. Rubel JA, Zilcha-Mano S, Giesemann J, Prinz J, Lutz W. Predicting personalized process-outcome associations in psychotherapy using machine learning approaches—A demonstration. Psychotherapy Research. 2020 Apr 2;30(3):300-9.

5. Niles AN, Loerinc AG, Krull JL, Roy-Byrne P, Sullivan G, Sherbourne CD, Bystritsky A, Craske MG. Advancing personalized medicine: application of a novel statistical method to identify treatment moderators in the coordinated anxiety learning and management study. Behavior therapy. 2017 Jul 1;48(4):490-500.

6. Higgins ET, Friedman RS, Harlow RE, Idson LC, Ayduk ON, Taylor A. Achievement orientations from subjective histories of success: Promotion pride versus prevention pride. European Journal of Social Psychology. 2001 Jan;31(1):3-23.

7. Lockwood P, Jordan CH, Kunda Z. Motivation by positive or negative role models: regulatory focus determines who will best inspire us. Journal of personality and social psychology. 2002 Oct;83(4):854.

8. Cacioppo JT, Petty RE, Feng Kao C. The efficient assessment of need for cognition. Journal of personality assessment. 1984 Jun 1;48(3):306-7.

10. Hadar L, Sood S. When knowledge is demotivating: subjective knowledge and choice overload. Psychological science. 2014 Sep;25(9):1739-47.

11. Choi I, Koo M, Choi JA. Individual differences in analytic versus holistic thinking. Personality and social psychology bulletin. 2007 May;33(5):691-705.

12. Vallacher RR, Wegner DM. Levels of personal agency: Individual variation in action identification. Journal of Personality and Social psychology. 1989 Oct;57(4):660.

13. Nenkov GY, Morrin M, Ward A, Schwartz B, Hulland J. A short form of the Maximization Scale: Factor structure, reliability and validity studies. Judgment and Decision Making. 2008 Jun;3(5):371-88.

14. Misuraca R, Faraci P, Gangemi A, Carmeci FA, Miceli S. The Decision Making Tendency Inventory: A new measure to assess maximizing, satisficing, and minimizing. Personality and Individual Differences. 2015 Oct 1;85:111-6.

15. Benoit ID, Miller EG. The mitigating role of holistic thinking on choice overload. Journal of Consumer Marketing. 2017 May 8;34(3):181-90.

16. Polman E. Effects of self–other decision making on regulatory focus and choice overload. Journal of personality and social psychology. 2012 May;102(5):980.

17. Diehl K, Poynor C. Great expectations?! Assortment size, expectations, and satisfaction. Journal of marketing research. 2010 Apr;47(2):312-22.

18. Holm S. A simple sequentially rejective multiple test procedure. Scandinavian journal of statistics. 1979 Jan 1:65-70.
